# Mother infant cortisol levels and maternal childhood adversity

**DOI:** 10.1038/s41598-025-28548-8

**Published:** 2025-12-29

**Authors:** Aline Camargo Ramos, Hugo Cogo-Moreira, Michael Eid, Vinicius Oliveira Santana, Lucas Pinto Ribeiro, Ana Carolina Coelho Milani, Ivaldo Silva, Cristiane S. Duarte, Jonathan Posner, Andrea Parolin Jackowski

**Affiliations:** 1 Laboratory of Integrative Neuroscience (LiNC), Universidade Federal de São Paulo, Rua Pedro de Toledo 669, 3O. Andar, São Paulo, SP Brazil; 2 Department of Psychiatry, Universidade Federal de São Paulo, São Paulo, Brazil; 3 Department of Education, Information and Communications Technology (ICT) and Learning, Østfold University College, Halden, Norway; 4 Department of Education and Psychology, Freie Universität Berlin, Berlin, Germany; 5 Department of Gynaecology, Universidade Federal de São Paulo, São Paulo, Brazil; 6 New York State Psychiatric Institute, Columbia University Irving Medical Center, New York, USA; 7 Department of Psychiatry & Behavioral Sciences, Duke University Medical Center, Durham, USA

**Keywords:** Childhood adversities, Cortisol, Attunement, Mother, Infant, HPA axis, Development of the nervous system, Stress and resilience

## Abstract

Cortisol, a key biomarker of hypothalamus–pituitary–adrenal (HPA) axis activity, is central to early stress regulation and neurodevelopment. While prior studies have linked maternal and infant cortisol to child outcomes, less is known about their synchrony during early infancy, a time of rapid neuroendocrine development. In this longitudinal study, we examined cortisol coupling and the correlation with maternal adverse childhood experiences (ACEs) in 305 mother–infant dyads from São Paulo, Brazil. Salivary cortisol was collected at ~ 1 month (32.3 days) and 6 months postpartum. We assessed intra- and interindividual cortisol dynamics and coupling using bivariate latent change score modeling. Maternal and infant cortisol were positively correlated at baseline (r = 0.319, p < 0.001) and at 6 months (r = 0.208, p = 0.003), suggesting early attunement that diminishes over time. Mothers and infants showed negative self-feedback, where higher baseline cortisol predicted smaller changes (mothers: B = -0.654; infants: B = -0.615; both p < 0.001). Maternal ACEs predicted elevated maternal cortisol at baseline (B = 0.126, p = 0.026) but did not affect the rate of change. These findings reveal early HPA synchrony and gradual decoupling, and highlight the lasting effects of maternal adversity on postpartum stress physiology.

**Supplementary Information:**

The online version contains supplementary material available at 10.1038/s41598-025-28548-8.

## Introduction

Adverse childhood events (ACEs) encompass physical, sexual, and psychological abuse, neglect, poverty, and parental mental illness^1^ and have been robustly linked to long-term alterations in mental health and behavior^2–6^. These early life adversities initiate neurobiological cascades that contribute to both functional and structural changes in brain regions critical for emotion regulation and stress response, including the prefrontal cortex, amygdala, and hippocampus^7–11^. Such alterations may underlie the psycho-emotional vulnerabilities observed in individuals with high ACE exposure and shape physiological regulation across generations. Building on this framework, the current study investigates (1) whether maternal and infant cortisol levels are linked during early infancy and (2) whether maternal ACEs relate to postpartum cortisol regulation and mother–infant synchrony.

The effects of ACEs extend beyond the directly affected individual and can impact the next generation, resulting in the intergenerational transmission of trauma^12–16^. This transmission can be associated with offspring development from the gestational period through postpartum, increasing the risk of mental disorders and metabolic diseases, among other outcomes^14,17,18^. The mechanisms underlying the intergenerational transmission of trauma are diverse, including genetic, hormonal, intrauterine environment, and neurobiological alterations^15,19,20^.

The impacts of ACEs on the hypothalamic–pituitary–adrenal (HPA) axis, one of the body’s central stress-response systems, are well established^21–26^. The HPA axis orchestrates physiological responses to threat and supports homeostasis by releasing corticotropin-releasing hormone (CRH), which stimulates adrenocorticotropic hormone (ACTH) secretion from the pituitary. ACTH then promotes cortisol release from the adrenal cortex, enabling broad systemic effects on metabolism, immunity, and neurodevelopment^27–29^. Dysregulation of this axis, as frequently observed in individuals exposed to early adversity, has implications for lifelong stress reactivity and health outcomes.

Stressors, such as unpredictability, uncontrollability, and social evaluation, increase cortisol levels, which typically return to baseline once the stressor subsides^30–32^. In contrast, ACEs are linked to chronic dysregulation of the HPA axis – in both hypo- or hyper-regulation^22^, affecting cortisol regulation across the lifespan^33^. According to Hosseini-Kamkar and colleagues^34^, HPA axis calibration follows an inverted U-shaped curve, with severe adversity leading to downregulation and milder stress to increased cortisol output. ACEs are also related with heightened HPA axis sensitivity during adulthood^21^. Women with elevated lifetime cortisol levels maintain higher levels during pregnancy, potentially increasing fetal exposure to stress^17,24,35^.

Under typical conditions, fetal exposure to maternal cortisol is regulated by the placental 11β-hydroxysteroid dehydrogenase type 2 (11β-HSD2) enzyme, which inactivates cortisol to cortisone, buffering the fetus from maternal glucocorticoid fluctuations^36,37^. However, 11β-HSD2 expression is reduced in pregnancies affected by elevated psychosocial stress, anxiety, or depression^38^ and this down regulation appears more pronounced in women with a history of ACEs, potentially increasing fetal cortisol exposure earlier in gestation^39^.

Glucocorticoids, such as cortisol, play a significant role in the development of the central nervous system, affecting emotional regulation and cognitive function. Physiological increases in cortisol levels during pregnancy are necessary for normative brain development and behavioral regulation^40^. Conversely, excessive exposure to cortisol during critical developmental periods appears to be detrimental to fetal neurodevelopment, with strong links to psychopathology later in life^3–6,41–43^.

Postnatally, maternal cortisol remains critical for infant development^44^, with cortisol related to cortisol regulation and temperament^45–47^. Children’s stress-regulatory systems are still developing, and caregivers play an important role in externally regulating their child’s stress response and helping them respond adaptively to stress and challenge^48^. Maternal exposure to ACEs has been associated with lower breast milk cortisol at 6 weeks and a blunted increase from weeks 2 to 12^49^, suggesting greater cortisol dysregulation as a function of an adverse environment^50^. However, research on circulating cortisol has yielded different results. Studies found that experiencing early life adversity was associated with alterations in diurnal cortisol rhythms across pregnancy^51,52^. Additionally, women who reported early life adversity had a larger cortisol awakening response than those who did not experience adversity at 2–6 months postpartum^53^. These findings underscore the complex role of maternal ACEs and HPA axis function and regulation, particularly during pregnancy and early postpartum, in the intergenerational transmission of adversities and may contribute to the fetal programming of infant stress physiology. Studies found that experiencing early life adversity was associated with alterations in diurnal cortisol rhythms across pregnancy^51,52^, and women reporting experiencing early life adversity had a larger awakening response than those who did not experience adversity at 2–6 months postpartum^53^. These findings underscore the role of maternal ACEs and HPA axis function and regulation, particularly during pregnancy and early postpartum, in the intergenerational transmission of adversities and may contribute to the fetal programming of infant stress physiology.

Physiological synchrony is a concept referring to the association between a specific measure of physiological activity in a caregiver and the same measure in their child, typically in response to a task or in a particular context. This core idea is measured and discussed using various related terms, such as concordance, attunement, coregulation, and coordination^54–56^. Within this framework, "synchrony," "concordance," and “attunement” generally denote a positive relationship where physiological activity moves in the same direction for both individuals. Conversely, terms like "negative attunement," "discordance," and “asynchrony” specifically denote a negative association where one individual’s physiological activity increases while the other’s decreases^48^.

Neuroendocrine, immune, and metabolic systems mediate the impact of maternal mental health on offspring, in physiological synchrony within the mother–infant dyad^57^. During pregnancy and the neonatal period, shared environments promote alignment of HPA axis activity between mothers and their children^58,59^. Studies have shown attunement in cortisol levels and diurnal rhythms within dyads, particularly between mothers and infants aged 7.8 to 17.4 months^60^. Correlations in cortisol were stronger for mother–infant pairs than father–infant pairs^61^, likely due to genetic, environmental, and emotional proximity. Maternal affective states are mirrored in infant physiology, while infant cues reciprocally are linked to maternal regulation, supporting a dynamic biobehavioral feedback system within the dyad^62^. This physiological synchrony and intergenerational transfer of adversity are hypothesized to be fundamentally mediated by epigenetic mechanisms. Specifically, epigenetic mechanisms, such as DNA methylation and changes in chromatin structure, have been implicated as a means by which environmental factors, like ACEs and stress, can shape gene expression and are thought to produce long-term health consequences in mothers and offspring^63,64^. In rats, stress situations have epigenetic effects that alter the methylation status of the NGFI-A binding site in a region of the Nr3c1 promoter responsible for control of hippocampal glucocorticoid receptor (GR) expression, and, in sum, results in decreased methylation of the NGFI-A consensus binding site, increased negative HPA feedback stress regulation^64^. In this way, our group found a negative association between maternal ACEs and miRNA expression (hsa-miR-582-3p) in umbilical cord blood samples^65^. High expression of hsa-miR-582-3p has been shown to negatively regulate the HPA axis through the modulation of glucocorticoid receptors^66^, and consequently, lower levels of this miRNA in newborns could lead to higher cortisol levels.

Biobehavioral synchrony reflects coordinated HPA axis activity within the mother–infant dyad in response to stress, and is thought to represent a biological expression of shared emotional and behavioral states^67–70^. While physiological attunement is commonly associated with positive outcomes, it may also occur in dysregulated forms. Mothers and infants can synchronize around both adaptive and maladaptive stress responses^71^. Specifically, parent–child physiological synchrony is beneficial by providing a scaffold from which children can develop independent regulatory skills^72,73^. Conversely, synchronous physiological activity can also be detrimental by potentially exaggerating the experience of stress between two individuals^74,75^. For instance, Fuchs (2017)^76^ found that cortisol awakening response (CAR) synchrony was present only in mother–child dyads with a history of maltreatment. These findings suggest that maternal ACEs may contribute to this maladaptive form of physiological attunement, thereby increasing the likelihood of intergenerational transmission of dysregulated stress physiology^19,20,43,77–79^.

Evidence suggests that HPA axis attunement may begin prenatally, with maternal cortisol levels during pregnancy accounting for approximately 30% of the variability in fetal cortisol^80^. Elevated maternal cortisol, particularly in the morning and afternoon, has been associated with increased infant cortisol reactivity and impaired stress recovery postnatally^81–83^. This physiological synchrony persists into the postpartum period, maintained through continuous behavioral and biological interactions^84^. However, maternal histories of childhood maltreatment have been linked to disrupted regulation in offspring, including lower baseline cortisol^43^ and prolonged elevated cortisol responses to stress^85^. These dyads also show reduced cortisol concordance, suggesting that early adversity in mothers may impair the transmission of adaptive stress regulation to the next generation^43,79,85^.

Cortisol levels among family members reflect not only genetic factors but also shared environmental factors^86^. As infants grow, their stress-response systems stabilize, and early mother–child interactions help shape enduring patterns of HPA axis regulation^87^. During infancy, cortisol is modulated by social input, particularly maternal behavior^50,88^. However, as children form relationships beyond the home, the maternal role wanes. The preschool period represents a sensitive window for cortisol consolidation and the development of stress regulation and resilience^87^. Thus, analyses of maternal-infant HPA axis dynamics must account for developmental stage, as regulatory patterns evolve rapidly over the first year of life^87^.

While prior research has demonstrated physiological attunement between maternal and infant cortisol levels, most studies have focused on dyads with infants older than 12 months^67,68^, with limited attention to earlier developmental stages (younger than 5 months)^85,89^. To address this gap, the present study examines mother–infant salivary cortisol attunement at 1 and 6 months postpartum—a period marked by rapid maturation of the infant stress-response system. We also assess changes in cortisol over time and evaluate the correlation between maternal exposure to ACEs and cortisol regulation in both mothers and their infants.

We hypothesize that maternal and infant cortisol levels will be positively correlated, consistent with early physiological attunement. We further hypothesize that greater maternal ACE exposure will be associated with disrupted cortisol regulation in the dyad (both mother and infant) during early infancy, which will in turn affect the development and stress response of the infant HPA axis.

## Methods

### Participants

Participants were drawn from the Mother Influences on Child Neurobehavioral Development (Healthy MiNDS) cohort study in Brazil^90^, a longitudinal study investigating the effects of maternal ACEs on offspring behavior, neurodevelopment, and associated biological mechanisms underlying intergenerational transmission. Maternal inclusion criteria were: residence in high-risk, low-resource areas of Guarulhos or São Paulo (São Paulo state, Brazil); reliance on the Brazilian public healthcare system (Sistema Único de Saúde); age between 18 and 38 years; gestational age of 25–39 weeks at enrollment; and the ability to read, understand, and provide written informed consent.

Mothers were excluded if they presented with high-risk pregnancies; severe psychiatric disorders (e.g., schizophrenia, bipolar disorder, persistent delusional disorder, obsessive–compulsive disorder, dementia, or suicidal ideation); history of traumatic brain injury, epilepsy treatment, or neurosurgery; decompensated medical conditions requiring intensive care; use of illicit substances (excluding cannabis); or active infections including toxoplasmosis, rubella, cytomegalovirus, herpes, or others.

Participants were enrolled at birth following maternal consent to participate in the study. Offspring were excluded if they met any of the following criteria: prematurity (< 37 weeks’ gestation), low birth weight (< 2.5 kg), 5-min Apgar score < 7, admission to a neonatal intensive care unit, or diagnosis of kernicterus or congenital metabolic disorders. The final sample comprised 325 mother–infant dyads recruited from the low-risk population of the Healthy MiNDS cohort.

This research protocol received approval from the Institutional Review Board (IRB) at Duke University (Pro00110664) and Columbia University/New York State Psychiatric Institute (7927). In Brazil, it was sanctioned by the National Research Ethics Committee (CONEP; 78,018,417.2.0000.5505) and the Research Ethics Committee of Universidade Federal de São Paulo (CEP; 1200/2017). All procedures were conducted in compliance with applicable guidelines and regulations.

Each participant in the study received comprehensive information regarding the study’s objectives and methodologies. Written informed consent was obtained for participation, including for their newborns, following the principles established in the Helsinki Declaration.

### Procedures

#### Maternal ACEs, race, maternal educational level, and socioeconomic status

Maternal ACEs were assessed during pregnancy using the CDC-Kaiser ACE Study Questionnaire^1,91^, administered at enrollment. This instrument evaluates adverse experiences before the age of 18 through 10 dichotomous items (“yes”/“no”), covering physical, emotional, and sexual abuse, neglect, and household dysfunction (e.g., domestic violence, substance use, mental illness, or parental separation). The total ACE score, ranging from 0 to 10, is the sum of affirmative responses, with higher scores reflecting greater cumulative exposure to childhood adversity.

Participants were asked to self-report their race, their infant’s race, and their educational attainment. Socioeconomic status was assessed using the ABEP^92^ (Associação Brasileira de Empresas de Pesquisa) scale, the official method for socioeconomic stratification in Brazil, which is based on household income, ownership of goods, and educational level of the head of household. The ABEP scale produces a total score ranging from 0 to 50, with classes approximately distributed as follows: A1 (45–50; $4745.29; 0.6%), A2 (41–44; value not reported), B1 (36–40; $2094.47; 1.1%), B2 (31–35; $1047.62; 7.9%), C1 (26–30; $572.96; 21.1%), C2 (15–25; $324.70; 36.0%), and D/E (0–14; $133.66; 33.4%). These values illustrate the socioeconomic diversity of the participants, spanning the range from the highest-income and highest-score class (A1) to the lowest-income and lowest-score groups (D/E).

#### Saliva sample collection

Saliva samples for cortisol measurement were collected from mothers and infants between 2–6 weeks postpartum (baseline) in the morning (7:00 am-12:00 pm) following their arrival at the university. Follow-up samples were collected when the infants reached 6 months of age, both in the morning and afternoon (8:30 a.m. to 6:00 p.m.), at the participants’ home.

Cortisol follows a circadian rhythm, peaking 20–30 min after awakening (the cortisol awakening response—CAR) and declining throughout the day to its lowest level 2–3 h after sleep onset^93^. Notably, Khoury^85^ found that saliva collection time was negatively correlated with maternal cortisol levels, but not with infant cortisol levels.

The saliva was collected using Salivette® (Sarstedt) tubes, placing the cotton swab under the tongue for 2–3 min. Mothers provided samples in Falcon tubes. All samples were centrifuged at 4,000 g for 15 min, aliquoted into microtubes, and stored in −80 °C freezers until the analysis.

### Measures

#### Measurement of salivary cortisol level

The enzyme-linked Immunosorbent Assay (ELISA) (DRG® Salivary Cortisol ELISA – SLV-2930 – MARBURG/Germany) was used to measure salivary cortisol levels based on the principle of competitive binding. This method utilizes wells coated with a monoclonal (mouse) antibody that targets an antigenic site on the cortisol molecule. Endogenous cortisol from the sample competes with a cortisol-peroxidase conjugate for binding to the antibody. After incubation, any unbound conjugate was washed away. The amount of peroxidase conjugate bound was inversely proportional to the cortisol level in the sample. Following the addition of the substrate solution, the intensity of the developed color was inversely proportional to the cortisol level in the sample. The results were presented as “absorbance obtained” and “final concentration of cortisol” in the analyzed sample.

### Statistical analysis

As an initial analysis, ordinary paired t-tests (6 months vs. baseline and mother vs. offspring) were conducted, along with Pearson correlations and partial correlations, to examine the nature of association within and between dyads over time. Standardized effect sizes are reported according to Cohen^94^, with the effect size for paired t-tests calculated using the standard deviation of the differences.

Subsequently, using the mother–offspring dyad data, the bivariate latent change score (BLCS) model was employed to investigate the concept of cross-domain coupling^95^. This model allowed us to assess the extent to which changes in salivary cortisol levels in offspring after 6 months (such as Δ [6 months − baseline]) are correlated with the initial maternal salivary cortisol level. Additionally, it quantified the changes in the salivary cortisol levels of both the offspring and mother between baseline and 6 months and their relationship with their respective baseline levels.

BLCS model with two-time points is a just-identified model fitting the data perfectly with zero degrees of freedom; therefore, no fit indices are reported. Detailed theoretical background on the BLCS model is provided in earlier works^96,97^, and Mplus and Lavaan syntaxes are presented in Supplementary Material 1.

**Fig. 1:**
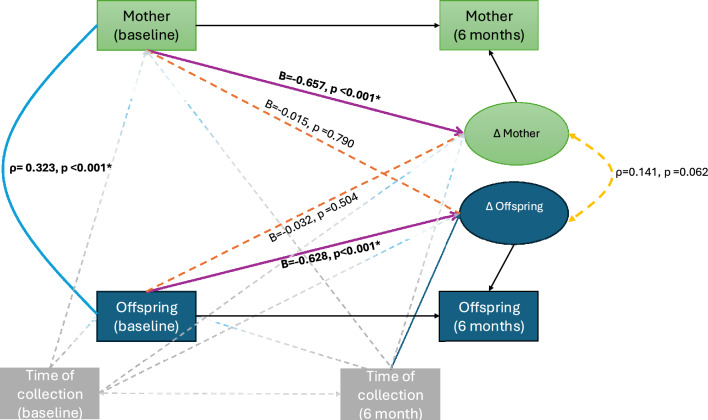
The main parameters derived from the BCLS model, including their standardized regression coefficients and respective p-values, are presented.

**Fig. 2:**
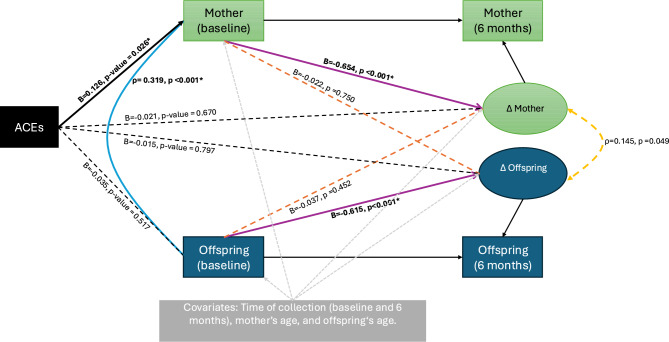
Effects of ACEs on the baseline and latent change measurements adjusted for time when cortisol was collected, mother’s and offspring’s age.

The main parameters of interest here are the following (presented in Fig. 1 and Fig. 2):

•The cross-domain coupling (shown in orange), represented by single-headed arrows.
•The self-feedback (shown in purple),
•The correlated change in cortisol levels (shown in yellow as a double-headed arrow), reflecting the degree to which changes in maternal and child salivary cortisol co-occur, after accounting for the coupling pathway.
•The mother-child correlation in cortisol levels at baseline (shown in blue as a double-headed arrow)

The raw values for the dyad cortisol were winsorized at the 5th and 95th percentile and log-transformed as conducted by Lawes^98^. Winsorization reduces the effect of extreme outliers by capping values at specified percentiles rather than removing them (unlike trimming), preserving the sample size (i.e., unlike deletion of outliers), reducing variance and skewness caused by extreme values. The log-transformation is used to normalize positively skewed data (common for hormone concentrations like cortisol) and stabilize variance. As a consequence, it makes the distribution more symmetric (closer to normality) and mitigates heteroscedasticity, facilitating linear modeling assumptions (e.g., regression).

Both raw and transformed salivary measures were presented, as the dispersion for offspring has not been described in the literature, to our knowledge.

Missing data were addressed using the full-information maximum likelihood approach based on the missing at-random mechanism^99^. Robust maximum likelihood estimation was applied because the transformed cortisol measures were not normally distributed.

Given the variation in the timing of cortisol data collection, and the known effect of cortisol levels based on the time of the day^100^, the BLCS model was adjusted to account for the timing of sample collection. This adjustment involves correlations between all initial cortisol measures and time, while also treating time as a predictor for the latent score change (see Supplementary Material 1 for the syntax and refer to the gray arrows and squares in Fig. 1).

Lastly, embedded in the BLCS model, we evaluated the correlation between ACEs and the cortisol baseline levels of the dyad and the latent change scores. Because this model is overidentified, we can assess its model fit (such as the comparative Fit Index, root mean squared error of approximation, and Tucker–Lewis index). We adopted the suggested cutoffs^101^ to determine whether the model’s fit was good or acceptable.

The racial diversity brought in the context of our study is not coming from a WEIRD (western, educated, industrialized, rich democracies) scenario^102^. Our hypothesis accounted for all variables, such as type of delivery and breastfeeding. These factors, among others, relate directly to the characterization of our study sample.

## Results

The mean maternal age was 27.30 years old (± 5.13), with an average of 3.53 ACEs. Infants were, on average, 32.32 days old at baseline and 203.22 days (approximately 6.77 months) at the 6-month follow-up. At least 39.5% of the mothers had completed high school. The majority of the sample (65.1%) identified as non-white, and 69.4% belonged to socioeconomic strata (classes DE and C2). More than half of the births were vaginal deliveries (61.4%). Detailed demographic characteristics are presented in Table 1.

**Table 1:** Maternal and Infant Demographics.

	**N**	**Minimum**	**Maximum**	**Mean/Count**	**Std. Deviation/%**
**INFANT**					
Age at baseline (days)	334	14	70	32.32	9.05
Age at 6-months (days)	203	157	255	203.22	18.6
**Sex**					
Female	181				50.60%
Male	177				49.40%
Birth weight (g)	347	2500	4500	3291.74	406.83
Birth height (cm)	332	43	56.50	48.76	1.85
**Type of birth**	334				
Vaginal delivery				205	61.4%
Cesarean section				125	37.4%
Forceps delivery				4	1.2%
**Feeding at baseline**	348				
Breastfeeding				252	72.4%
Formula				31	8.9%
Mixed (Breastfeeding + Formula)				65	
**MOTHER**					
Age (years)	358	18	38	27.30	5.13
ACEs	358	0	10	3.53	2.35
**Race and ethnicity**	358				
White				125	34.9%
Black				142	39.7%
Asian				63	17.6%
Mixed-race				27	7.5%
Indigenous				1	0.3%
**Socioeconomic class and status**	356				
U$ 4745,29 (A1)				2	0.6%
U$ 2094,47 (B1)				4	1.1%
U$ 1047,62 (B2)				28	7.9%
U$ 572,96 (C1)				75	21.1%
U$ 324,70 (C2)				128	36.0%
U$ 133,66 (DE)				119	33.4%
**Education**	357				
Completed elementary school				4	1.10%
Incomplete middle school				6	1.70%
Complete middle school				9	2.50%
Incomplete high school				65	18.20%
Complete high school				141	39.50%
Incomplete higher education				53	14.80%
Complete higher education				79	22.10%
**MATERNAL AND INFANT**					
Cortisol collection time (baseline)	325	7:05 AM	12:39 PM	9:39 AM	1:05
Cortisol collection time (6-months)	203	8:30 AM	6 PM	12:44 PM	2:05

From baseline to six months postpartum, salivary cortisol levels significantly declined in both mothers and their infants, with a greater reduction observed in maternal levels. For mothers, both raw (M = –0.381, 95% CI [–0.537, –0.224]) and winsorized log-transformed values (M = –0.433, 95% CI [–0.590, –0.274]) showed significant decreases. Similarly, infants exhibited significant reductions over time in both raw (M = –0.173, 95% CI [–0.319, –0.026]) and winsorized values (M = –0.195, 95% CI [–0.341, –0.048]). Full results are presented in Table 2.

**Table 2:** Descriptive statistics for the dyad’s cortisol levels (ng/mL) under the raw values and winsorized and log-transformed data.

	Cortisol Baseline Assessment					Cortisol After Six Months						
	N	Minimum	Maximum	Mean (ng/mL)	Std. Deviation	N	Minimum	Maximum	Mean (ng/mL)	Std. Deviation	Difference (Six months-Baseline) Paired t-test (Cohen’s d)	95% CI
Mother raw	305	0.1	18	3.59	3.48	203	0.271	17.11	2.31	2.27	−0.381	−0.537 to −0.224
Offspring raw	325	0.56	33.94	6.43	6.13	203	0.24	35.28	5.49	5.48	−0.173	−0.319 to −0.026
Mother wl	305	0.58	11.93	3.47	3.06	203	0.439	6.74	2.18	1.72	−0.433	−0.590 to −0.274
Offspring wl	325	1.14	20.44	6.23	5.42	203	0.671	17.43	5.29	4.77	−0.195	−0.341 to −0.048

Mother–offspring cortisol levels are positively correlated, with stronger associations observed at baseline (r = 0.319, p < 0.001) than at six months (r = 0.208, p = 0.003). At baseline, correlations were moderate and statistically significant, while at six months, the correlations weakened but remained statistically significant. Correlations were controlled for the time of saliva collection to account for diurnal variation in cortisol levels. Detailed correlation and p-values for both raw and winsorized data are provided in Table 3.

**Table 3:** Pearson correlations and partial correlations between mother–offspring dyads’ cortisol levels.

Dyad	Pearson correlation	p-value	Partial correlation	p-value
Mother–offspring (baseline) raw	0.319	< 0.001	0.371	< 0.001
Mother–offspring (baseline) wl	0.379	< 0.001	0.375	< 0.001
Mother–offspring (six months) raw	0.208	0.003	0.165	0.019
Mother–offspring (six months) wl	0.19	0.007	0.148	0.035

Standardized effect sizes (Cohen’s d) for differences in cortisol levels between mothers and their offspring at baseline and six months, based on both raw and winsorized (WL) data, are presented in Table 4. Across all conditions, offspring exhibited higher cortisol levels than their mothers.

**Table 4:** Standardized effect sizes (Cohen’s d and its lower and upper bounds) between the mother and offspring cortisol levels.

	Baseline (Mother–offspring)		Six months (Mother–offspring)	
	Raw	WL	Raw	WL
Mean	−2.856	−3.182	−2.760	−3.109
Std. Deviation	5.834	5.474	5.136	4.754
Cohen D	−0.488	−0.581	−0.537	−0.654
Cohen’s D Lower bound	−0.606	−0.729	−0.657	−0.805
Cohen’s D Upper bound	−0.369	−0.431	−0.417	−0.502

The figures present the four main parameters of interest in their standardized form (i.e., standardized regression coefficients and Pearson’s correlations) taking into account the time when the cortisol was collected for Fig. 1 and full model where ACEs’ baseline cortisol levels and latent change score are adjusted for time when cortisol was collected, mother’s and offspring’s age in Fig. 2. A significance level of 0.05 was adopted, and the significant paths are indicated using solid lines for easier visualization.

Under the full adjusted model and full-information maximum likelihood approach for dealing with missing data, the baseline correlation between mother–offspring cortisol levels was significant (r = 0.319, 95% CI = 0.212–0.425), indicating a medium-sized effect^94^. In contrast, the correlation between changes in cortisol levels over time within the dyad was smaller (r = 0.145, 95% CI = 0.001 to 0.289), and the difference between these two correlations was statistically significant (Wald test (1) = 4.664, p = 0.0308). These results suggest a stronger synchrony in cortisol levels at baseline compared to changes over time. See Fig. 1 and 2 for a visual representation of the BCLS model parameters (n = 358) without and with covariates adjustment, respectively.

ACEs significantly predicted maternal baseline cortisol levels (B = 0.126, p = 0.026), while all other paths from ACEs to cortisol outcomes were not statistically significant. Importantly, the effect sizes of the other model paths remained unchanged after including ACEs and covariates (mothers’ and offsprings’ age). The model was overidentified and demonstrated excellent fit (χ2(6) = 7.727, p = 0.2588; CFI = 0.981 and TLI = 0.915; RMSEA = 0.028, 90% CI = 0.000 to 0.078; RMSEA p = 0.705). These models fit and findings are derived from the model represented in Fig. 2.

**Fig. 3:**
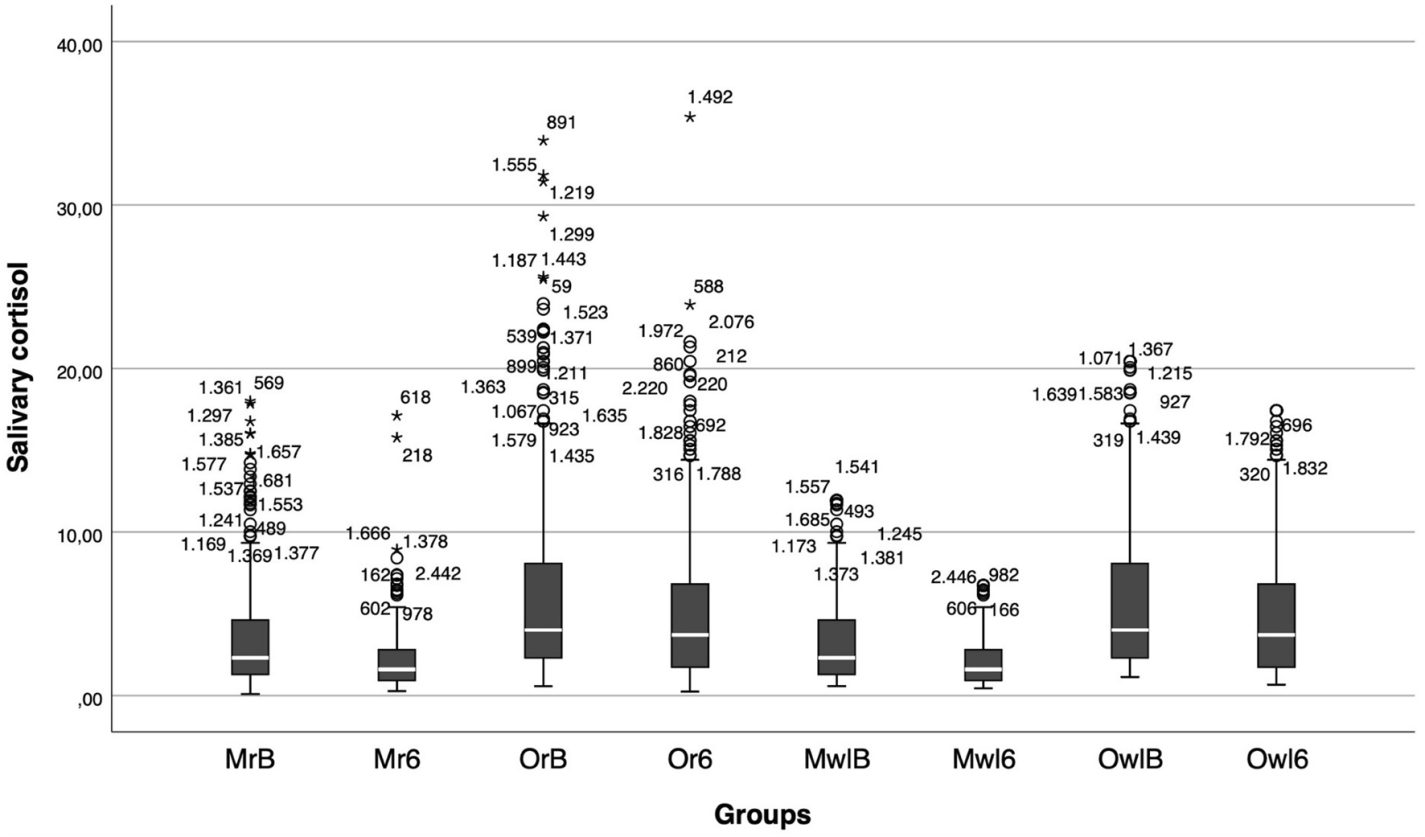
Box plots display the raw and winsorized cortisol values distribution for mothers and their offspring at baseline and six months postpartum. Legend: MrB = Mother row baseline cortisol, Mr6 = mother raw six-month cortisol, OrB = offspring raw baseline cortisol, Or6 = offspring raw six months cortisol, MwlB = Mother winsorized and log-transformed baseline cortisol, Mwl6 = mother winsorized and log-transformed six-month cortisol, OwlB = offspring winsorized and log-transformed baseline cortisol, Owl6 = offspring winsorized and log-transformed six-months cortisol.

The raw and winsorized values of salivary cortisol for mothers and their offspring at baseline and at 6 months are presented in Fig. 3.

**Fig. 4:**
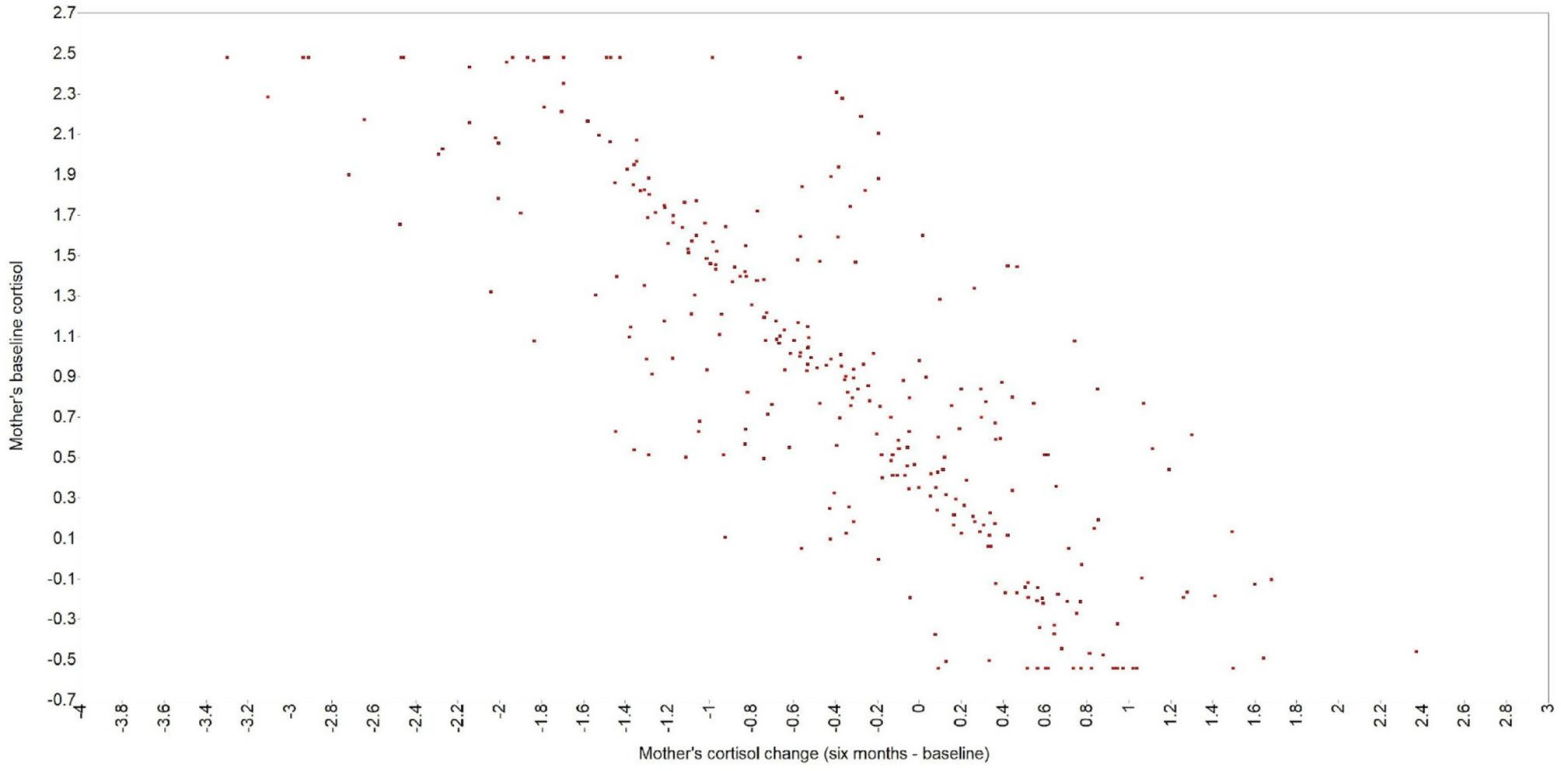
Scatterplot of the self-feedback for mothers’ cortisol.

Only the self-feedback paths were statistically significant and showed negative, similarly sized effects, indicating that higher baseline cortisol levels were associated with greater decreases over time for both mothers and offspring. Conversely, individuals with lower baseline cortisol levels showed increases over time. This pattern suggests a regulatory effect toward the mean in both groups (Fig. 4). In contrast, the cross-domain coupling paths were not statistically significant and had minimal effect sizes, indicating no meaningful correlation between one dyad member’s cortisol trajectory with the others.

Supplementary Material 1 presents all estimated unstandardized and standardized parameters, including their confidence intervals. It also details the factors that were included as covariates in our model: maternal age, ethnicity, education, SES, ACEs, and maternal mental health (GAD7, PHQ9, PSS, EPDS, and resilience). However, none of these covariates correlated with the presented results, except for maternal ACEs.

Consistent with expectations for longitudinal studies, the research experienced participant loss during data collection, with 344 dyads’ saliva samples collected at baseline and 203 samples retained at 6-month follow-up (243 dyads performed the collection, but in 40 cases there was insufficient saliva volume to perform the cortisol analysis). To assess potential attrition bias, we compared the profile of participants who completed the follow-up to those who did not. No significant differences were observed across key maternal characteristics, including age (mean 27.6 vs. 26.9 years; t = −1.38; p = 0.17), number of ACEs (mean 3.61 vs. 3.45; t = −0.61; p = 0.54), or SES score (mean 20.16 vs. 19.71; t = −0.63; p = 0.53). Likewise, the distribution of maternal race did not differ significantly (χ2 = 3.18; p = 0.53), and while a non-significant trend was observed for maternal education (χ2 = 13.92; p = 0.053), it was not statistically significant. These findings suggest that the participant loss at the 6-month follow-up did not introduce substantial bias in the main maternal characteristics, thereby supporting the representativeness of the retained sample. Important to note that the results derived from the full adjusted model analysed data from 350 dyads due to the full-information maximum likelihood approach, as described in the statistical analysis subheading.

## Discussion

Our findings indicate that maternal ACEs were associated with elevated salivary cortisol levels in mothers during the neonatal period, but this association was not sustained at six months postpartum. This temporal pattern may reflect the heightened physiological and psychological demands of the early postpartum period, particularly for women with a history of childhood adversity. Prior research suggests that early-life stress can recalibrate the HPA axis, increasing baseline activity and reactivity to stress^103,104^. Chronic exposure to elevated cortisol during development may disrupt glucocorticoid receptor sensitivity, resulting in long-term alterations to stress regulation^27^.

The first three months following birth involve significant lifestyle and circadian disruptions, including sleep deprivation, hormonal shifts associated with breastfeeding, and increased caregiving demands. These stressors may disproportionately impact mothers with elevated vulnerability due to ACEs, exacerbating HPA axis activation^47,49,53,105,106^. Circadian misalignment and sensitivity to daily stressors may contribute to transient elevations in maternal cortisol, particularly in those with heightened affective reactivity and limited regulatory capacity.

Interestingly, our results diverge from prior work showing blunted cortisol responses in mothers with higher ACE exposure^49^. However, that study examined a low-risk sample characterized by low adversity and substantial partner involvement, which may buffer the physiological effects of early adversity. Conversely, a study evaluating adolescent mothers from a high-risk Brazilian birth cohort project found that lifetime trauma history was associated with maternal salivary cortisol at 12 months postpartum^107^. Taken together, our findings underscore the importance of contextual and temporal factors in shaping the relationship between maternal ACEs and HPA axis activity during the postpartum period. Factors such as exposure to current stress, family environment, socioeconomic issues, and social support are crucial in mitigating or exacerbating the effects of early exposure to adversity on HPA axis regulation and cortisol levels^107,108^.

We observed a significant decline in maternal salivary cortisol concentrations from the neonatal period (3.59 ± 3.48 ng/mL) to six months postpartum (2.31 ± 2.27 ng/mL), consistent with established patterns of elevated cortisol during late pregnancy and during labor^109,110^. This trajectory reflects the physiological tapering of maternal HPA axis activity following childbirth.

Uniquely, our study examined mother-infant cortisol synchronicity at two critical developmental stages: 1) during the neonatal period (~ 1 month of age) and again at 6 months. While prior research has assessed maternal and infant cortisol levels independently, few studies have addressed their physiological coupling in the early postpartum months, and none, to our knowledge, have done so longitudinally from the neonatal stage onward. By capturing this dyadic cortisol dynamic across time, our findings provide novel insight into the temporal emergence and evolution of mother–infant HPA axis synchrony, a mechanism increasingly recognized as central to biobehavioral regulation in early development.

We observed a significant positive correlation between maternal and infant salivary cortisol levels at both the neonatal stage (r = 0.319, p < 0.001) and at 6 months postpartum (r = 0.208, p = 0.003), with stronger synchrony in the early postnatal period. This finding is consistent with evidence suggesting that mothers and infants exhibit physiological, emotional, and behavioral attunement from birth onward^59,62,111^. Such synchrony is thought to originate during gestation, when maternal and fetal systems are coupled through shared neuroendocrine pathways^112^. Maternal cortisol, for example, has been shown to correlate with levels in both amniotic fluid and fetal cord blood^113,114^, providing a route for the programming of infant stress responses. In line with this, Nazzari and coworkers^115^ reported that higher maternal cortisol awakening response (CAR) during pregnancy predicted a flatter infant cortisol response to stress in the neonatal period. However, findings remain mixed; in a later study, antenatal diurnal cortisol levels were not associated with stress reactivity in 3-month-old infants^116^. Collectively, these results suggest that maternal–infant physiological coupling is dynamic and may be strongest immediately after birth, potentially shaped by both prenatal exposures and early caregiving contexts.

The observed decrease in cortisol synchrony from the neonatal period to 6 months may reflect developmental changes following birth. As the direct physiological coupling between mother and fetus is disrupted postnatally, and as the infant’s central nervous system matures, dyadic cortisol attunement naturally declines. This pattern is consistent with prior evidence suggesting that cortisol synchrony is sustained not only by biological embedding but also by shared environments and co-regulated routines^61,86,117,118^. Notably, studies have found that parent–child adrenocortical synchrony depends on the amount of time spent together, underscoring the role of behavioral and contextual factors in shaping physiological alignment^61,117,118^.

Our sample comprised mothers with a mean of 3.6 ACEs, a rate notably higher than that reported in studies from high-income settings^119,120^. This elevated exposure to early adversity may partially account for the high level of dyadic cortisol attunement observed, particularly at baseline. Consistent with this interpretation, Hibel and collaborators^121^ reported that mothers and infants exposed to family violence exhibited greater physiological synchrony during stress, compared to non-exposed dyads. Similarly, heightened cortisol concordance has been observed in dyads facing elevated psychosocial risk or environmental threat^67^. Furthermore, our findings align with those from a São Paulo birth cohort project, a high-risk group characterized by chronic stress and residents primarily living in slums. Liu and colleagues^108^ found a strong correlation between maternal and infant hair cortisol sampled at 12 months in this cohort. The high degree of maternal-infant cortisol synchrony observed in these two high-risk studies, relative to findings from other mother-infant studies, strongly suggests that context plays an important role in the synchrony of maternal and infant cortisol. However, this association may be non-linear. Khoury^85^ found that maternal history of childhood maltreatment moderated the relationship between maternal and infant cortisol: while low-maltreatment dyads exhibited positive cortisol associations, high-maltreatment dyads did not. Interestingly, in that study, maternal and infant cortisol levels remained highly correlated at 4 months of age, suggesting that developmental timing and maternal history interact to shape the dynamics of HPA axis co-regulation. Our findings align with this literature, indicating that maternal early-life adversity may enhance mother–infant physiological synchrony during early infancy, particularly under conditions of elevated caregiving demands. However, in high-risk circumstances (such as those involving high socioeconomic risk, maltreatment, or insecure attachment), synchrony was not associated with better interaction quality or child functioning. This suggests that, under conditions of elevated risk, synchrony may instead act as an additional risk factor in the intergenerational transmission of the effects of stress and trauma^122^.

Finally, we investigated whether baseline cortisol levels in mothers and their infants predicted their own cortisol levels six months later, focusing on intraindividual change, termed here as self-feedback. We found that higher baseline cortisol levels were associated with greater reductions over time in both mothers and infants, whereas lower initial levels tended to increase.

These findings align with established models of HPA axis regulation, in which cortisol secretion activates a negative feedback loop through glucocorticoid receptor (GR) binding, restoring baseline levels following stress exposure^123^. During pregnancy, however, sustained activation of the HPA axis results in elevated cortisol, which typically normalizes postpartum^124^.

Under chronic stress, reduced GR sensitivity can impair this negative feedback mechanism, leading to HPA dysregulation and glucocorticoid resistance^125^. While our data revealed typical feedback patterns in cortisol recovery across the postpartum period, maternal ACEs did not significantly moderate this self-feedback dynamic. This suggests that ACE-related disruptions in HPA function may be more pronounced in acute stress reactivity than in basal feedback processes. However, our results do indicate that ACEs correlates with maternal cortisol concentrations during the neonatal period, consistent with the hypothesis that early adversity sensitizes the HPA axis, rendering it more reactive to environmental stressors encountered later in life^126^. In line with this, mothers with early trauma have shown elevated basal cortisol levels across the day, potentially reflecting a hyperresponsive HPA axis^53^. Importantly, while we focused on cortisol as an index of HPA function, other mechanisms – including ACTH levels and epigenetic modifications of GR expression – likely contribute to the regulation of this system and warrant future investigation.

Our results should also be interpreted in light of several limitations. First, maternal ACEs were assessed retrospectively, introducing potential recall bias. Second, our design relied on naturally occurring variation in cortisol levels rather than experimental stress paradigms, which limits causal inference.

Statistically, although it is possible to test a BLCS model with a minimum of two time points, but there are critics of this use given that two time point models, like the latent change score here presented are unlikely to capture individual differences reliably because of relatively little shared variance with the true generating scores and lower reliabilities^127^; consequently there is an attenuation of relationships that individual trajectories would exhibit with other phenomena^128^. Some insights and suggestions for two-time points are given by Brandmaier^129^, where some factors are study duration and the influence of adding an extra measurement occasion could influence the precision to estimate individual differences in linear slopes; however, there are always practicalities and logistics related to such precision gain as costs and adherence of the participants.

Lastly, there was inherent variation in the timing of saliva collection for both mothers and infants, which may have affected the measured cortisol values. This timing variability was primarily observed in the six-month follow-up collections, which were performed at the participants’ homes, according to their provided schedule. To mitigate these effects, collection time was included as a covariate in the statistical analyses.

Despite these limitations, our study offers several strengths. We included a racially and socioeconomically diverse cohort of mothers, predominantly Black and mixed-race women from under-resourced urban communities in Brazil, a population often underrepresented in developmental psychobiology. In this context, it is known that exposure to childhood adversity affects racial minorities and low-income communities, who are more likely to experience systemic and community adversities^130^. Moreover, we assessed mother-infant cortisol levels at two critical time points—during the neonatal period and at six months postpartum—an approach rarely undertaken in prior studies. Most prior research on maternal and infant cortisol has focused on post-stress reactivity, often using maternal self-collection at home^51,115,116,131–135^. In contrast, our data were collected at rest and under controlled conditions by trained researchers, ensuring procedural consistency across all participants. The resulting cortisol values are in line with those reported in previous studies^43,111,116,136,137^, supporting the validity and robustness of our protocol.

In summary, our findings highlight the enduring role of maternal ACEs, particularly in the context of early parenting and biological attunement with the infant. We demonstrated that maternal early adversity is associated with elevated cortisol in the neonatal period and that maternal and infant cortisol levels are significantly synchronized at early stages of development. These patterns highlight potential pathways for intergenerational transmission of stress biology and suggest critical windows for intervention to promote long-term health and developmental outcomes in vulnerable populations.

## Supplementary Information

Supplementary Information.

## Data Availability

The raw data supporting the findings of this study are available from the corresponding author (AJ) upon request. Raw data are not publicly available due to restrictions in the written consent signed by the participants of our study.

## Abbreviations

ACEs: Adverse childhood experiences
HPA: Hypothalamic–pituitary–adrenal
