# Supplementary material for: Mother infant cortisol levels and maternal childhood adversity
Source: Sci Rep. 2025 Dec 29;15:44746. doi: 10.1038/s41598-025-28548-8 (PMC12749534; doi:10.1038/s41598-025-28548-8)
Supplement: Supplementary file 1 — Supplementary Information. [file 41598_2025_28548_MOESM1_ESM.docx]

Supplementary Material 1

Mplus VERSION 8.7

MUTHEN & MUTHEN

05/28/2024 10:07 AM

INPUT INSTRUCTIONS

Title: Change score aline;

Data: file is Matrix_time_winso.dat;

Variable: names are ID age etnicity schooling ABEP NmerodeACEs

GAD7SCORERM GAD7SCOREFUP6M

PHQ9SCORERM PHQ9SCOREFUP6M PSSSCOREBASELINE PSSSCOREFUP6M

EPDSSCORE RESILIENCIASCORE m_cort_rm

m_cort_6m M_ot age_aname etnicity_baby Sex Delivery Feed_rm

Feed_6m Birthweight Height_born

head_born Weight_rm Height_rm head_rm WEIGHT6M HEIGHT6M

head_6m baby_ot b_cort_rm b_cort_6m time_d

Time_6m_d time_rm_c time_6m_c m_cort_rm_w m_cort_6m_w

b_cort_rm_w b_cort_6m_w idn;

missing are all (-99);

usevariable are time_rm_c time_6m_c x1 x3 y1 y3 ;

Define:

x1 = log(m_cort_rm_w);!MOTHER CORTISOL BASELINE!

x3 = log(m_cort_6m_w);!MOTHER CORTISOL 6 MONTHS!

y1 = log(b_cort_rm_w);!BABY COSRTISOL BASELINE!

y3 = log(b_cort_6m_w);!BABY CORTISOL 6 MOTNHS!

ANALYSIS:

MODEL = NOCOV; ! Sets all default covariances to zero

Estimator = MLR;

MODEL:

x3 ON x1@1;

! This parameter regresses COG_T2 perfectly on COG_T1

dx BY x3@1;

! This defines the latent change score factor as measured perfectly by scores on COG

[dx];

! This estimates the intercept of the change score

[x1];

! This estimates the intercept of COG_T1

[x3@0];

! This constrains the intercept of COG_T2 to 0

! LCS model for NEU

y3 ON y1@1;

! This parameter regresses NEU_T2 perfectly on NEU_T1

dy BY y3@1;

! This defines the latent change score factor as measured perfectly by scores on NE

[y3@0];

! This line constrains the intercept of NEU_T2 to 0

y3@0;

! This fixes the variance of the NEU_T1 to 0

! Parameters estimated in the model

dx; !dy;

! This estimates the variance of the change scores

x1;

! This estimates the variance of the COG_T1

x3@0;

! This fixes the variance of the COG_T2 to 0

[dy];

! This estimates the intercept of the change score

[y1];

! This estimates the intercept of NEU_T1

dy;

! This estimates the variance of the change scores

y1;

! This estimates the variance of NEU_T1

dy ON x1 y1;

! This estimates the COG to NEU coupling parameter and the COG to COG self-feedback

dx ON y1 x1;

! This estimates the NEU to COG coupling parameter and the NEU to NEU self-feedback

x1 WITH y1 (a);

! This estimates the COG_T1 NEU_T1 covariance

dx with dy (b);

! This estimates the dx and dy covariance

dx dy on time_6m_c time_rm_c;

x1 y1 with time_rm_c time_6m_c;

time_6m_c with time_rm_c;

Model test:

0 = a-b;

OUTPUT: CINTERVAL STANDARDIZED;

plot: type = plot3;

*** WARNING

Input line exceeded 90 characters. Some input may be truncated.

! This defines the latent change score factor as measured perfectly by scores on COG_

*** WARNING

Input line exceeded 90 characters. Some input may be truncated.

! This defines the latent change score factor as measured perfectly by scores on NEU

*** WARNING in VARIABLE command

Note that only the first 8 characters of variable names are used in the output.

Shorten variable names to avoid any confusion.

*** WARNING in PLOT command

Note that only the first 8 characters of variable names are used in plots.

If variable names are not unique within the first 8 characters, problems

may occur.

*** WARNING

Data set contains cases with missing on all variables.

These cases were not included in the analysis.

Number of cases with missing on all variables: 12

5 WARNING(S) FOUND IN THE INPUT INSTRUCTIONS

Change score aline;

SUMMARY OF ANALYSIS

Number of groups 1

Number of observations 346

Number of dependent variables 2

Number of independent variables 4

Number of continuous latent variables 2

Observed dependent variables

Continuous

X3 Y3

Observed independent variables

TIME_RM_ TIME_6M_ X1 Y1

Continuous latent variables

DX DY

Estimator MLR

Information matrix OBSERVED

Maximum number of iterations 1000

Convergence criterion 0.500D-04

Maximum number of steepest descent iterations 20

Maximum number of iterations for H1 2000

Convergence criterion for H1 0.100D-03

Input data file(s)

Matrix_time_winso.dat

Input data format FREE

SUMMARY OF DATA

Number of missing data patterns 5

COVARIANCE COVERAGE OF DATA

Minimum covariance coverage value 0.100

PROPORTION OF DATA PRESENT

Covariance Coverage

X3 Y3 TIME_RM_ TIME_6M_ X1

________ ________ ________ ________ ________

X3 0.587

Y3 0.587 0.587

TIME_RM_ 0.526 0.526 0.939

TIME_6M_ 0.587 0.587 0.526 0.587

X1 0.486 0.486 0.882 0.486 0.882

Y1 0.526 0.526 0.939 0.526 0.882

Covariance Coverage

Y1

________

Y1 0.939

UNIVARIATE SAMPLE STATISTICS

UNIVARIATE HIGHER-ORDER MOMENT DESCRIPTIVE STATISTICS

Variable/ Mean/ Skewness/ Minimum/ % with Percentiles

Sample Size Variance Kurtosis Maximum Min/Max 20%/60% 40%/80% Median

X3 0.498 0.150 -0.823 5.42% -0.202 0.239 0.470

203.000 0.566 -0.817 1.908 5.42% 0.647 1.131

Y3 1.273 -0.047 -0.399 5.42% 0.445 1.075 1.311

203.000 0.838 -0.795 2.858 5.42% 1.548 2.059

TIME_RM_C 9.699 1.382 7.083 0.31% 8.717 9.167 9.583

325.000 1.550 4.153 15.617 0.31% 9.833 10.583

TIME_6M_C 12.744 0.539 8.500 0.49% 10.750 11.583 12.000

203.000 4.507 -0.630 19.417 0.49% 13.083 14.733

X1 0.892 0.131 -0.545 5.25% 0.122 0.610 0.837

305.000 0.716 -0.840 2.479 5.25% 1.065 1.696

Y1 1.487 0.191 0.131 5.23% 0.688 1.233 1.389

325.000 0.685 -0.896 3.017 5.23% 1.688 2.232

THE MODEL ESTIMATION TERMINATED NORMALLY

MODEL FIT INFORMATION

Number of Free Parameters 27

Loglikelihood

H0 Value -2213.191

H0 Scaling Correction Factor 0.9899

for MLR

H1 Value -2213.191

H1 Scaling Correction Factor 0.9899

for MLR

Information Criteria

Akaike (AIC) 4480.383

Bayesian (BIC) 4584.237

Sample-Size Adjusted BIC 4498.585

(n* = (n + 2) / 24)

Chi-Square Test of Model Fit

Value 0.000*

Degrees of Freedom 0

P-Value 0.0000

Scaling Correction Factor 1.0000

for MLR

* The chi-square value for MLM, MLMV, MLR, ULSMV, WLSM and WLSMV cannot be used

for chi-square difference testing in the regular way. MLM, MLR and WLSM

chi-square difference testing is described on the Mplus website. MLMV, WLSMV,

and ULSMV difference testing is done using the DIFFTEST option.

RMSEA (Root Mean Square Error Of Approximation)

Estimate 0.000

90 Percent C.I. 0.000 0.000

Probability RMSEA <= .05 0.000

CFI/TLI

CFI 1.000

TLI 1.000

Chi-Square Test of Model Fit for the Baseline Model

Value 40.476

Degrees of Freedom 9

P-Value 0.0000

SRMR (Standardized Root Mean Square Residual)

Value 0.000

Wald Test of Parameter Constraints

Value 5.027

Degrees of Freedom 1

P-Value 0.0250

MODEL RESULTS

Two-Tailed

Estimate S.E. Est./S.E. P-Value

DX BY

X3 1.000 0.000 999.000 999.000

DY BY

Y3 1.000 0.000 999.000 999.000

DY ON

X1 -0.022 0.082 -0.266 0.790

Y1 -0.904 0.077 -11.770 0.000

TIME_6M_C -0.048 0.029 -1.692 0.091

TIME_RM_C 0.031 0.050 0.620 0.535

DX ON

Y1 -0.042 0.063 -0.668 0.504

X1 -0.837 0.065 -12.841 0.000

TIME_6M_C -0.122 0.021 -5.705 0.000

TIME_RM_C 0.084 0.034 2.436 0.015

X3 ON

X1 1.000 0.000 999.000 999.000

Y3 ON

Y1 1.000 0.000 999.000 999.000

DX WITH

DY 0.089 0.048 1.847 0.065

X1 WITH

Y1 0.225 0.042 5.408 0.000

TIME_RM_C -0.090 0.057 -1.572 0.116

TIME_6M_C 0.311 0.152 2.041 0.041

Y1 WITH

TIME_RM_C -0.132 0.059 -2.232 0.026

TIME_6M_C 0.111 0.133 0.833 0.405

TIME_6M_ WITH

TIME_RM_C 0.030 0.172 0.172 0.864

Means

TIME_RM_C 9.697 0.069 140.856 0.000

TIME_6M_C 12.726 0.148 86.251 0.000

X1 0.892 0.048 18.554 0.000

Y1 1.487 0.046 32.387 0.000

Intercepts

X3 0.000 0.000 999.000 999.000

Y3 0.000 0.000 999.000 999.000

DX 1.149 0.446 2.578 0.010

DY 1.462 0.632 2.312 0.021

Variances

TIME_RM_C 1.550 0.213 7.271 0.000

TIME_6M_C 4.506 0.369 12.213 0.000

X1 0.713 0.044 16.262 0.000

Y1 0.685 0.040 17.162 0.000

Residual Variances

X3 0.000 0.000 999.000 999.000

Y3 0.000 0.000 999.000 999.000

DX 0.486 0.040 12.181 0.000

DY 0.821 0.065 12.596 0.000

QUALITY OF NUMERICAL RESULTS

Condition Number for the Information Matrix 0.188E-04

(ratio of smallest to largest eigenvalue)

STANDARDIZED MODEL RESULTS

STDYX Standardization

Two-Tailed

Estimate S.E. Est./S.E. P-Value

DX BY

X3 1.428 0.074 19.177 0.000

DY BY

Y3 1.302 0.056 23.382 0.000

DY ON

X1 -0.015 0.058 -0.266 0.790

Y1 -0.628 0.038 -16.343 0.000

TIME_6M_C -0.086 0.051 -1.693 0.090

TIME_RM_C 0.032 0.052 0.622 0.534

DX ON

Y1 -0.032 0.048 -0.668 0.504

X1 -0.657 0.036 -18.460 0.000

TIME_6M_C -0.240 0.043 -5.551 0.000

TIME_RM_C 0.097 0.040 2.412 0.016

X3 ON

X1 1.121 0.053 21.008 0.000

Y3 ON

Y1 0.905 0.045 20.215 0.000

DX WITH

DY 0.141 0.075 1.865 0.062

X1 WITH

Y1 0.323 0.054 5.928 0.000

TIME_RM_C -0.085 0.053 -1.598 0.110

TIME_6M_C 0.173 0.083 2.093 0.036

Y1 WITH

TIME_RM_C -0.128 0.056 -2.300 0.021

TIME_6M_C 0.063 0.075 0.842 0.400

TIME_6M_ WITH

TIME_RM_C 0.011 0.065 0.172 0.864

Means

TIME_RM_C 7.790 0.507 15.361 0.000

TIME_6M_C 5.995 0.224 26.731 0.000

X1 1.056 0.062 16.988 0.000

Y1 1.796 0.069 26.029 0.000

Intercepts

X3 0.000 0.000 999.000 999.000

Y3 0.000 0.000 999.000 999.000

DX 1.069 0.407 2.624 0.009

DY 1.227 0.523 2.345 0.019

Variances

TIME_RM_C 1.000 0.000 999.000 999.000

TIME_6M_C 1.000 0.000 999.000 999.000

X1 1.000 0.000 999.000 999.000

Y1 1.000 0.000 999.000 999.000

Residual Variances

X3 0.000 999.000 999.000 999.000

Y3 0.000 999.000 999.000 999.000

DX 0.420 0.042 10.018 0.000

DY 0.578 0.047 12.312 0.000

STDY Standardization

Two-Tailed

Estimate S.E. Est./S.E. P-Value

DX BY

X3 1.428 0.074 19.177 0.000

DY BY

Y3 1.302 0.056 23.382 0.000

DY ON

X1 -0.015 0.058 -0.266 0.790

Y1 -0.628 0.038 -16.343 0.000

TIME_6M_C -0.086 0.051 -1.693 0.090

TIME_RM_C 0.032 0.052 0.622 0.534

DX ON

Y1 -0.032 0.048 -0.668 0.504

X1 -0.657 0.036 -18.460 0.000

TIME_6M_C -0.240 0.043 -5.551 0.000

TIME_RM_C 0.097 0.040 2.412 0.016

X3 ON

X1 1.121 0.053 21.008 0.000

Y3 ON

Y1 0.905 0.045 20.215 0.000

DX WITH

DY 0.141 0.075 1.865 0.062

X1 WITH

Y1 0.323 0.054 5.928 0.000

TIME_RM_C -0.085 0.053 -1.598 0.110

TIME_6M_C 0.173 0.083 2.093 0.036

Y1 WITH

TIME_RM_C -0.128 0.056 -2.300 0.021

TIME_6M_C 0.063 0.075 0.842 0.400

TIME_6M_ WITH

TIME_RM_C 0.011 0.065 0.172 0.864

Means

TIME_RM_C 7.790 0.507 15.361 0.000

TIME_6M_C 5.995 0.224 26.731 0.000

X1 1.056 0.062 16.988 0.000

Y1 1.796 0.069 26.029 0.000

Intercepts

X3 0.000 0.000 999.000 999.000

Y3 0.000 0.000 999.000 999.000

DX 1.069 0.407 2.624 0.009

DY 1.227 0.523 2.345 0.019

Variances

TIME_RM_C 1.000 0.000 999.000 999.000

TIME_6M_C 1.000 0.000 999.000 999.000

X1 1.000 0.000 999.000 999.000

Y1 1.000 0.000 999.000 999.000

Residual Variances

X3 999.000 999.000 999.000 999.000

Y3 999.000 999.000 999.000 999.000

DX 0.420 0.042 10.018 0.000

DY 0.578 0.047 12.312 0.000

STD Standardization

Two-Tailed

Estimate S.E. Est./S.E. P-Value

DX BY

X3 1.075 0.052 20.489 0.000

DY BY

Y3 1.191 0.053 22.305 0.000

DY ON

X1 -0.018 0.069 -0.266 0.790

Y1 -0.758 0.044 -17.164 0.000

TIME_6M_C -0.041 0.024 -1.703 0.088

TIME_RM_C 0.026 0.042 0.621 0.535

DX ON

Y1 -0.039 0.058 -0.669 0.503

X1 -0.778 0.042 -18.375 0.000

TIME_6M_C -0.113 0.020 -5.749 0.000

TIME_RM_C 0.078 0.032 2.423 0.015

X3 ON

X1 1.000 0.000 999.000 999.000

Y3 ON

Y1 1.000 0.000 999.000 999.000

DX WITH

DY 0.141 0.075 1.865 0.062

X1 WITH

Y1 0.225 0.042 5.408 0.000

TIME_RM_C -0.090 0.057 -1.572 0.116

TIME_6M_C 0.311 0.152 2.041 0.041

Y1 WITH

TIME_RM_C -0.132 0.059 -2.232 0.026

TIME_6M_C 0.111 0.133 0.833 0.405

TIME_6M_ WITH

TIME_RM_C 0.030 0.172 0.172 0.864

Means

TIME_RM_C 9.697 0.069 140.856 0.000

TIME_6M_C 12.726 0.148 86.251 0.000

X1 0.892 0.048 18.554 0.000

Y1 1.487 0.046 32.387 0.000

Intercepts

X3 0.000 0.000 999.000 999.000

Y3 0.000 0.000 999.000 999.000

DX 1.069 0.407 2.624 0.009

DY 1.227 0.523 2.345 0.019

Variances

TIME_RM_C 1.550 0.213 7.271 0.000

TIME_6M_C 4.506 0.369 12.213 0.000

X1 0.713 0.044 16.262 0.000

Y1 0.685 0.040 17.162 0.000

Residual Variances

X3 999.000 999.000 999.000 999.000

Y3 999.000 999.000 999.000 999.000

DX 0.420 0.042 10.018 0.000

DY 0.578 0.047 12.312 0.000

R-SQUARE

Observed Two-Tailed

Variable Estimate S.E. Est./S.E. P-Value

X3 1.000 999.000 999.000 999.000

Y3 1.000 999.000 999.000 999.000

Latent Two-Tailed

Variable Estimate S.E. Est./S.E. P-Value

DX 0.580 0.042 13.845 0.000

DY 0.422 0.047 8.983 0.000

CONFIDENCE INTERVALS OF MODEL RESULTS

Lower .5% Lower 2.5% Lower 5% Estimate Upper 5% Upper 2.5% Upper .5%

DX BY

X3 1.000 1.000 1.000 1.000 1.000 1.000 1.000

DY BY

Y3 1.000 1.000 1.000 1.000 1.000 1.000 1.000

DY ON

X1 -0.233 -0.183 -0.157 -0.022 0.113 0.139 0.190

Y1 -1.101 -1.054 -1.030 -0.904 -0.777 -0.753 -0.706

TIME_6M_C -0.122 -0.105 -0.096 -0.048 -0.001 0.008 0.025

TIME_RM_C -0.098 -0.067 -0.051 0.031 0.113 0.129 0.159

DX ON

Y1 -0.203 -0.165 -0.145 -0.042 0.061 0.081 0.120

X1 -1.005 -0.964 -0.944 -0.837 -0.730 -0.709 -0.669

TIME_6M_C -0.177 -0.164 -0.157 -0.122 -0.087 -0.080 -0.067

TIME_RM_C -0.005 0.016 0.027 0.084 0.140 0.151 0.172

X3 ON

X1 1.000 1.000 1.000 1.000 1.000 1.000 1.000

Y3 ON

Y1 1.000 1.000 1.000 1.000 1.000 1.000 1.000

DX WITH

DY -0.035 -0.005 0.010 0.089 0.168 0.183 0.212

X1 WITH

Y1 0.118 0.144 0.157 0.225 0.294 0.307 0.333

TIME_RM_C -0.236 -0.201 -0.183 -0.090 0.004 0.022 0.057

TIME_6M_C -0.081 0.012 0.060 0.311 0.561 0.609 0.703

Y1 WITH

TIME_RM_C -0.285 -0.248 -0.229 -0.132 -0.035 -0.016 0.020

TIME_6M_C -0.231 -0.150 -0.108 0.111 0.329 0.371 0.453

TIME_6M_ WITH

TIME_RM_C -0.415 -0.308 -0.254 0.030 0.313 0.368 0.474

Means

TIME_RM_ 9.520 9.562 9.584 9.697 9.810 9.832 9.875

TIME_6M_ 12.346 12.437 12.484 12.726 12.969 13.016 13.106

X1 0.768 0.798 0.813 0.892 0.971 0.986 1.016

Y1 1.369 1.397 1.411 1.487 1.562 1.577 1.605

Intercepts

X3 0.000 0.000 0.000 0.000 0.000 0.000 0.000

Y3 0.000 0.000 0.000 0.000 0.000 0.000 0.000

DX 0.001 0.276 0.416 1.149 1.882 2.023 2.297

DY -0.167 0.223 0.422 1.462 2.502 2.701 3.090

Variances

TIME_RM_ 1.001 1.132 1.199 1.550 1.900 1.968 2.099

TIME_6M_ 3.556 3.783 3.899 4.506 5.113 5.230 5.457

X1 0.600 0.627 0.641 0.713 0.785 0.799 0.826

Y1 0.583 0.607 0.620 0.685 0.751 0.764 0.788

Residual Variances

X3 0.000 0.000 0.000 0.000 0.000 0.000 0.000

Y3 0.000 0.000 0.000 0.000 0.000 0.000 0.000

DX 0.383 0.407 0.420 0.486 0.551 0.564 0.588

DY 0.653 0.693 0.713 0.821 0.928 0.948 0.988

CONFIDENCE INTERVALS OF STANDARDIZED MODEL RESULTS

STDYX Standardization

Lower .5% Lower 2.5% Lower 5% Estimate Upper 5% Upper 2.5% Upper .5%

DX BY

X3 1.236 1.282 1.306 1.428 1.551 1.574 1.620

DY BY

Y3 1.159 1.193 1.210 1.302 1.394 1.411 1.445

DY ON

X1 -0.165 -0.129 -0.111 -0.015 0.080 0.099 0.134

Y1 -0.727 -0.703 -0.691 -0.628 -0.565 -0.553 -0.529

TIME_6M_C -0.218 -0.186 -0.170 -0.086 -0.002 0.014 0.045

TIME_RM_C -0.102 -0.070 -0.053 0.032 0.118 0.134 0.166

DX ON

Y1 -0.157 -0.127 -0.112 -0.032 0.047 0.062 0.092

X1 -0.749 -0.727 -0.715 -0.657 -0.598 -0.587 -0.565

TIME_6M_C -0.352 -0.325 -0.311 -0.240 -0.169 -0.155 -0.129

TIME_RM_C -0.007 0.018 0.031 0.097 0.163 0.176 0.201

X3 ON

X1 0.984 1.017 1.033 1.121 1.209 1.226 1.259

Y3 ON

Y1 0.790 0.817 0.831 0.905 0.978 0.993 1.020

DX WITH

DY -0.054 -0.007 0.017 0.141 0.265 0.288 0.335

X1 WITH

Y1 0.182 0.216 0.233 0.323 0.412 0.429 0.463

TIME_RM_C -0.223 -0.190 -0.173 -0.085 0.003 0.019 0.052

TIME_6M_C -0.040 0.011 0.037 0.173 0.310 0.336 0.387

Y1 WITH

TIME_RM_C -0.272 -0.237 -0.220 -0.128 -0.037 -0.019 0.015

TIME_6M_C -0.130 -0.084 -0.060 0.063 0.186 0.209 0.255

TIME_6M_ WITH

TIME_RM_C -0.157 -0.117 -0.096 0.011 0.119 0.139 0.179

Means

TIME_RM_ 6.483 6.796 6.955 7.790 8.624 8.784 9.096

TIME_6M_ 5.417 5.555 5.626 5.995 6.364 6.435 6.573

X1 0.896 0.934 0.954 1.056 1.159 1.178 1.217

Y1 1.618 1.661 1.683 1.796 1.910 1.931 1.974

Intercepts

X3 0.000 0.000 0.000 0.000 0.000 0.000 0.000

Y3 0.000 0.000 0.000 0.000 0.000 0.000 0.000

DX 0.020 0.271 0.399 1.069 1.738 1.867 2.117

DY -0.121 0.201 0.366 1.227 2.088 2.253 2.575

Variances

TIME_RM_ 1.000 1.000 1.000 1.000 1.000 1.000 1.000

TIME_6M_ 1.000 1.000 1.000 1.000 1.000 1.000 1.000

X1 1.000 1.000 1.000 1.000 1.000 1.000 1.000

Y1 1.000 1.000 1.000 1.000 1.000 1.000 1.000

Residual Variances

X3 999.000 999.000 999.000 0.000 999.000 999.000 999.000

Y3 999.000 999.000 999.000 0.000 999.000 999.000 999.000

DX 0.312 0.338 0.351 0.420 0.489 0.502 0.528

DY 0.457 0.486 0.501 0.578 0.655 0.670 0.699

STDY Standardization

Lower .5% Lower 2.5% Lower 5% Estimate Upper 5% Upper 2.5% Upper .5%

DX BY

X3 1.236 1.282 1.306 1.428 1.551 1.574 1.620

DY BY

Y3 1.159 1.193 1.210 1.302 1.394 1.411 1.445

DY ON

X1 -0.165 -0.129 -0.111 -0.015 0.080 0.099 0.134

Y1 -0.727 -0.703 -0.691 -0.628 -0.565 -0.553 -0.529

TIME_6M_C -0.218 -0.186 -0.170 -0.086 -0.002 0.014 0.045

TIME_RM_C -0.102 -0.070 -0.053 0.032 0.118 0.134 0.166

DX ON

Y1 -0.157 -0.127 -0.112 -0.032 0.047 0.062 0.092

X1 -0.749 -0.727 -0.715 -0.657 -0.598 -0.587 -0.565

TIME_6M_C -0.352 -0.325 -0.311 -0.240 -0.169 -0.155 -0.129

TIME_RM_C -0.007 0.018 0.031 0.097 0.163 0.176 0.201

X3 ON

X1 0.984 1.017 1.033 1.121 1.209 1.226 1.259

Y3 ON

Y1 0.790 0.817 0.831 0.905 0.978 0.993 1.020

DX WITH

DY -0.054 -0.007 0.017 0.141 0.265 0.288 0.335

X1 WITH

Y1 0.182 0.216 0.233 0.323 0.412 0.429 0.463

TIME_RM_C -0.223 -0.190 -0.173 -0.085 0.003 0.019 0.052

TIME_6M_C -0.040 0.011 0.037 0.173 0.310 0.336 0.387

Y1 WITH

TIME_RM_C -0.272 -0.237 -0.220 -0.128 -0.037 -0.019 0.015

TIME_6M_C -0.130 -0.084 -0.060 0.063 0.186 0.209 0.255

TIME_6M_ WITH

TIME_RM_C -0.157 -0.117 -0.096 0.011 0.119 0.139 0.179

Means

TIME_RM_ 6.483 6.796 6.955 7.790 8.624 8.784 9.096

TIME_6M_ 5.417 5.555 5.626 5.995 6.364 6.435 6.573

X1 0.896 0.934 0.954 1.056 1.159 1.178 1.217

Y1 1.618 1.661 1.683 1.796 1.910 1.931 1.974

Intercepts

X3 0.000 0.000 0.000 0.000 0.000 0.000 0.000

Y3 0.000 0.000 0.000 0.000 0.000 0.000 0.000

DX 0.020 0.271 0.399 1.069 1.738 1.867 2.117

DY -0.121 0.201 0.366 1.227 2.088 2.253 2.575

Variances

TIME_RM_ 1.000 1.000 1.000 1.000 1.000 1.000 1.000

TIME_6M_ 1.000 1.000 1.000 1.000 1.000 1.000 1.000

X1 1.000 1.000 1.000 1.000 1.000 1.000 1.000

Y1 1.000 1.000 1.000 1.000 1.000 1.000 1.000

Residual Variances

X3 999.000 999.000 999.000 999.000 999.000 999.000 999.000

Y3 999.000 999.000 999.000 999.000 999.000 999.000 999.000

DX 0.312 0.338 0.351 0.420 0.489 0.502 0.528

DY 0.457 0.486 0.501 0.578 0.655 0.670 0.699

STD Standardization

Lower .5% Lower 2.5% Lower 5% Estimate Upper 5% Upper 2.5% Upper .5%

DX BY

X3 0.940 0.973 0.989 1.075 1.162 1.178 1.211

DY BY

Y3 1.054 1.087 1.103 1.191 1.279 1.296 1.329

DY ON

X1 -0.196 -0.153 -0.132 -0.018 0.095 0.117 0.159

Y1 -0.872 -0.845 -0.831 -0.758 -0.686 -0.672 -0.645

TIME_6M_C -0.102 -0.087 -0.080 -0.041 -0.001 0.006 0.021

TIME_RM_C -0.082 -0.056 -0.043 0.026 0.095 0.108 0.134

DX ON

Y1 -0.189 -0.153 -0.135 -0.039 0.057 0.075 0.111

X1 -0.887 -0.861 -0.848 -0.778 -0.708 -0.695 -0.669

TIME_6M_C -0.164 -0.152 -0.146 -0.113 -0.081 -0.075 -0.062

TIME_RM_C -0.005 0.015 0.025 0.078 0.131 0.141 0.161

X3 ON

X1 1.000 1.000 1.000 1.000 1.000 1.000 1.000

Y3 ON

Y1 1.000 1.000 1.000 1.000 1.000 1.000 1.000

DX WITH

DY -0.054 -0.007 0.017 0.141 0.265 0.288 0.335

X1 WITH

Y1 0.118 0.144 0.157 0.225 0.294 0.307 0.333

TIME_RM_C -0.236 -0.201 -0.183 -0.090 0.004 0.022 0.057

TIME_6M_C -0.081 0.012 0.060 0.311 0.561 0.609 0.703

Y1 WITH

TIME_RM_C -0.285 -0.248 -0.229 -0.132 -0.035 -0.016 0.020

TIME_6M_C -0.231 -0.150 -0.108 0.111 0.329 0.371 0.453

TIME_6M_ WITH

TIME_RM_C -0.415 -0.308 -0.254 0.030 0.313 0.368 0.474

Means

TIME_RM_ 9.520 9.562 9.584 9.697 9.810 9.832 9.875

TIME_6M_ 12.346 12.437 12.484 12.726 12.969 13.016 13.106

X1 0.768 0.798 0.813 0.892 0.971 0.986 1.016

Y1 1.369 1.397 1.411 1.487 1.562 1.577 1.605

Intercepts

X3 0.000 0.000 0.000 0.000 0.000 0.000 0.000

Y3 0.000 0.000 0.000 0.000 0.000 0.000 0.000

DX 0.020 0.271 0.399 1.069 1.738 1.867 2.117

DY -0.121 0.201 0.366 1.227 2.088 2.253 2.575

Variances

TIME_RM_ 1.001 1.132 1.199 1.550 1.900 1.968 2.099

TIME_6M_ 3.556 3.783 3.899 4.506 5.113 5.230 5.457

X1 0.600 0.627 0.641 0.713 0.785 0.799 0.826

Y1 0.583 0.607 0.620 0.685 0.751 0.764 0.788

Residual Variances

X3 999.000 999.000 999.000 999.000 999.000 999.000 999.000

Y3 999.000 999.000 999.000 999.000 999.000 999.000 999.000

DX 0.312 0.338 0.351 0.420 0.489 0.502 0.528

DY 0.457 0.486 0.501 0.578 0.655 0.670 0.699

SAMPLE STATISTICS FOR ESTIMATED FACTOR SCORES

SAMPLE STATISTICS

Means

DX DX_SE DY DY_SE

________ ________ ________ ________

-0.396 0.393 -0.218 0.427

Covariances

DX DX_SE DY DY_SE

________ ________ ________ ________

DX 0.855

DX_SE 0.015 0.147

DY 0.306 0.029 1.035

DY_SE 0.014 0.157 0.025 0.202

Correlations

DX DX_SE DY DY_SE

________ ________ ________ ________

DX 1.000

DX_SE 0.043 1.000

DY 0.325 0.074 1.000

DY_SE 0.034 0.912 0.054 1.000

PLOT INFORMATION

The following plots are available:

Histograms (sample values, estimated factor scores, estimated values, residuals)

Scatterplots (sample values, estimated factor scores, estimated values, residuals)

Latent variable distribution plots

DIAGRAM INFORMATION

Use View Diagram under the Diagram menu in the Mplus Editor to view the diagram.

If running Mplus from the Mplus Diagrammer, the diagram opens automatically.

Diagram output

c:\users\hugoc\dropbox\trabalhos\aline -posdoc\cortisol\time\correlation_difference_test.dgm

Beginning Time: 10:07:17

Ending Time: 10:07:17

Elapsed Time: 00:00:00

MUTHEN & MUTHEN

3463 Stoner Ave.

Los Angeles, CA 90066

Tel: (310) 391-9971

Fax: (310) 391-8971

Web: www.StatModel.com

Support: Support@StatModel.com

Copyright (c) 1998-2021 Muthen & Muthen

Mplus VERSION 8.7

MUTHEN & MUTHEN

05/28/2024 9:49 AM

INPUT INSTRUCTIONS

Title: Change score aline;

Data: file is Matrix_time_winso.dat;

Variable: names are ID age etnicity schooling ABEP NmerodeACEs

GAD7SCORERM GAD7SCOREFUP6M

PHQ9SCORERM PHQ9SCOREFUP6M PSSSCOREBASELINE PSSSCOREFUP6M

EPDSSCORE RESILIENCIASCORE m_cort_rm

m_cort_6m M_ot age_aname etnicity_baby Sex Delivery Feed_rm

Feed_6m Birthweight Height_born

head_born Weight_rm Height_rm head_rm WEIGHT6M HEIGHT6M

head_6m baby_ot b_cort_rm b_cort_6m time_d

Time_6m_d time_rm_c time_6m_c m_cort_rm_w m_cort_6m_w

b_cort_rm_w b_cort_6m_w idn;

missing are all (-99);

usevariable are time_rm_c time_6m_c x1 x3 y1 y3 ;

Define:

x1 = log(m_cort_rm_w);!MOTHER CORTISOL BASELINE!

x3 = log(m_cort_6m_w);!MOTHER CORTISOL 6 MONTHS!

y1 = log(b_cort_rm_w);!BABY COSRTISOL BASELINE!

y3 = log(b_cort_6m_w);!BABY CORTISOL 6 MOTNHS!

ANALYSIS:

MODEL = NOCOV; ! Sets all default covariances to zero

Estimator = MLR;

MODEL:

x3 ON x1@1;

! This parameter regresses COG_T2 perfectly on COG_T1

dx BY x3@1;

! This defines the latent change score factor as measured perfectly by scores on COG

[dx];

! This estimates the intercept of the change score

[x1];

! This estimates the intercept of COG_T1

[x3@0];

! This constrains the intercept of COG_T2 to 0

! LCS model for NEU

y3 ON y1@1;

! This parameter regresses NEU_T2 perfectly on NEU_T1

dy BY y3@1;

! This defines the latent change score factor as measured perfectly by scores on NE

[y3@0];

! This line constrains the intercept of NEU_T2 to 0

y3@0;

! This fixes the variance of the NEU_T1 to 0

! Parameters estimated in the model

dx; !dy;

! This estimates the variance of the change scores

x1;

! This estimates the variance of the COG_T1

x3@0;

! This fixes the variance of the COG_T2 to 0

[dy];

! This estimates the intercept of the change score

[y1];

! This estimates the intercept of NEU_T1

dy;

! This estimates the variance of the change scores

y1;

! This estimates the variance of NEU_T1

dy ON x1 y1;

! This estimates the COG to NEU coupling parameter and the COG to COG self-feedback

dx ON y1 x1;

! This estimates the NEU to COG coupling parameter and the NEU to NEU self-feedback

x1 WITH y1;

! This estimates the COG_T1 NEU_T1 covariance

dx with dy;

! This estimates the dx and dy covariance

dx dy on time_6m_c time_rm_c;

x1 y1 with time_rm_c time_6m_c;

time_6m_c with time_rm_c;

OUTPUT: CINTERVAL STANDARDIZED;

plot: type = plot3;

*** WARNING

Input line exceeded 90 characters. Some input may be truncated.

! This defines the latent change score factor as measured perfectly by scores on COG_

*** WARNING

Input line exceeded 90 characters. Some input may be truncated.

! This defines the latent change score factor as measured perfectly by scores on NEU

*** WARNING in VARIABLE command

Note that only the first 8 characters of variable names are used in the output.

Shorten variable names to avoid any confusion.

*** WARNING in PLOT command

Note that only the first 8 characters of variable names are used in plots.

If variable names are not unique within the first 8 characters, problems

may occur.

*** WARNING

Data set contains cases with missing on all variables.

These cases were not included in the analysis.

Number of cases with missing on all variables: 12

5 WARNING(S) FOUND IN THE INPUT INSTRUCTIONS

Change score aline;

SUMMARY OF ANALYSIS

Number of groups 1

Number of observations 346

Number of dependent variables 2

Number of independent variables 4

Number of continuous latent variables 2

Observed dependent variables

Continuous

X3 Y3

Observed independent variables

TIME_RM_ TIME_6M_ X1 Y1

Continuous latent variables

DX DY

Estimator MLR

Information matrix OBSERVED

Maximum number of iterations 1000

Convergence criterion 0.500D-04

Maximum number of steepest descent iterations 20

Maximum number of iterations for H1 2000

Convergence criterion for H1 0.100D-03

Input data file(s)

Matrix_time_winso.dat

Input data format FREE

SUMMARY OF DATA

Number of missing data patterns 5

COVARIANCE COVERAGE OF DATA

Minimum covariance coverage value 0.100

PROPORTION OF DATA PRESENT

Covariance Coverage

X3 Y3 TIME_RM_ TIME_6M_ X1

________ ________ ________ ________ ________

X3 0.587

Y3 0.587 0.587

TIME_RM_ 0.526 0.526 0.939

TIME_6M_ 0.587 0.587 0.526 0.587

X1 0.486 0.486 0.882 0.486 0.882

Y1 0.526 0.526 0.939 0.526 0.882

Covariance Coverage

Y1

________

Y1 0.939

UNIVARIATE SAMPLE STATISTICS

UNIVARIATE HIGHER-ORDER MOMENT DESCRIPTIVE STATISTICS

Variable/ Mean/ Skewness/ Minimum/ % with Percentiles

Sample Size Variance Kurtosis Maximum Min/Max 20%/60% 40%/80% Median

X3 0.498 0.150 -0.823 5.42% -0.202 0.239 0.470

203.000 0.566 -0.817 1.908 5.42% 0.647 1.131

Y3 1.273 -0.047 -0.399 5.42% 0.445 1.075 1.311

203.000 0.838 -0.795 2.858 5.42% 1.548 2.059

TIME_RM_C 9.699 1.382 7.083 0.31% 8.717 9.167 9.583

325.000 1.550 4.153 15.617 0.31% 9.833 10.583

TIME_6M_C 12.744 0.539 8.500 0.49% 10.750 11.583 12.000

203.000 4.507 -0.630 19.417 0.49% 13.083 14.733

X1 0.892 0.131 -0.545 5.25% 0.122 0.610 0.837

305.000 0.716 -0.840 2.479 5.25% 1.065 1.696

Y1 1.487 0.191 0.131 5.23% 0.688 1.233 1.389

325.000 0.685 -0.896 3.017 5.23% 1.688 2.232

THE MODEL ESTIMATION TERMINATED NORMALLY

MODEL FIT INFORMATION

Number of Free Parameters 27

Loglikelihood

H0 Value -2213.191

H0 Scaling Correction Factor 0.9899

for MLR

H1 Value -2213.191

H1 Scaling Correction Factor 0.9899

for MLR

Information Criteria

Akaike (AIC) 4480.383

Bayesian (BIC) 4584.237

Sample-Size Adjusted BIC 4498.585

(n* = (n + 2) / 24)

Chi-Square Test of Model Fit

Value 0.000*

Degrees of Freedom 0

P-Value 0.0000

Scaling Correction Factor 1.0000

for MLR

* The chi-square value for MLM, MLMV, MLR, ULSMV, WLSM and WLSMV cannot be used

for chi-square difference testing in the regular way. MLM, MLR and WLSM

chi-square difference testing is described on the Mplus website. MLMV, WLSMV,

and ULSMV difference testing is done using the DIFFTEST option.

RMSEA (Root Mean Square Error Of Approximation)

Estimate 0.000

90 Percent C.I. 0.000 0.000

Probability RMSEA <= .05 0.000

CFI/TLI

CFI 1.000

TLI 1.000

Chi-Square Test of Model Fit for the Baseline Model

Value 40.476

Degrees of Freedom 9

P-Value 0.0000

SRMR (Standardized Root Mean Square Residual)

Value 0.000

MODEL RESULTS

Two-Tailed

Estimate S.E. Est./S.E. P-Value

DX BY

X3 1.000 0.000 999.000 999.000

DY BY

Y3 1.000 0.000 999.000 999.000

DY ON

X1 -0.022 0.082 -0.266 0.790

Y1 -0.904 0.077 -11.770 0.000

TIME_6M_C -0.048 0.029 -1.692 0.091

TIME_RM_C 0.031 0.050 0.620 0.535

DX ON

Y1 -0.042 0.063 -0.668 0.504

X1 -0.837 0.065 -12.841 0.000

TIME_6M_C -0.122 0.021 -5.705 0.000

TIME_RM_C 0.084 0.034 2.436 0.015

X3 ON

X1 1.000 0.000 999.000 999.000

Y3 ON

Y1 1.000 0.000 999.000 999.000

DX WITH

DY 0.089 0.048 1.847 0.065

X1 WITH

Y1 0.225 0.042 5.408 0.000

TIME_RM_C -0.090 0.057 -1.572 0.116

TIME_6M_C 0.311 0.152 2.041 0.041

Y1 WITH

TIME_RM_C -0.132 0.059 -2.232 0.026

TIME_6M_C 0.111 0.133 0.833 0.405

TIME_6M_ WITH

TIME_RM_C 0.030 0.172 0.172 0.864

Means

TIME_RM_C 9.697 0.069 140.856 0.000

TIME_6M_C 12.726 0.148 86.251 0.000

X1 0.892 0.048 18.554 0.000

Y1 1.487 0.046 32.387 0.000

Intercepts

X3 0.000 0.000 999.000 999.000

Y3 0.000 0.000 999.000 999.000

DX 1.149 0.446 2.578 0.010

DY 1.462 0.632 2.312 0.021

Variances

TIME_RM_C 1.550 0.213 7.271 0.000

TIME_6M_C 4.506 0.369 12.213 0.000

X1 0.713 0.044 16.262 0.000

Y1 0.685 0.040 17.162 0.000

Residual Variances

X3 0.000 0.000 999.000 999.000

Y3 0.000 0.000 999.000 999.000

DX 0.486 0.040 12.181 0.000

DY 0.821 0.065 12.596 0.000

QUALITY OF NUMERICAL RESULTS

Condition Number for the Information Matrix 0.188E-04

(ratio of smallest to largest eigenvalue)

STANDARDIZED MODEL RESULTS

STDYX Standardization

Two-Tailed

Estimate S.E. Est./S.E. P-Value

DX BY

X3 1.428 0.074 19.177 0.000

DY BY

Y3 1.302 0.056 23.382 0.000

DY ON

X1 -0.015 0.058 -0.266 0.790

Y1 -0.628 0.038 -16.343 0.000

TIME_6M_C -0.086 0.051 -1.693 0.090

TIME_RM_C 0.032 0.052 0.622 0.534

DX ON

Y1 -0.032 0.048 -0.668 0.504

X1 -0.657 0.036 -18.460 0.000

TIME_6M_C -0.240 0.043 -5.551 0.000

TIME_RM_C 0.097 0.040 2.412 0.016

X3 ON

X1 1.121 0.053 21.008 0.000

Y3 ON

Y1 0.905 0.045 20.215 0.000

DX WITH

DY 0.141 0.075 1.865 0.062

X1 WITH

Y1 0.323 0.054 5.928 0.000

TIME_RM_C -0.085 0.053 -1.598 0.110

TIME_6M_C 0.173 0.083 2.093 0.036

Y1 WITH

TIME_RM_C -0.128 0.056 -2.300 0.021

TIME_6M_C 0.063 0.075 0.842 0.400

TIME_6M_ WITH

TIME_RM_C 0.011 0.065 0.172 0.864

Means

TIME_RM_C 7.790 0.507 15.361 0.000

TIME_6M_C 5.995 0.224 26.731 0.000

X1 1.056 0.062 16.988 0.000

Y1 1.796 0.069 26.029 0.000

Intercepts

X3 0.000 0.000 999.000 999.000

Y3 0.000 0.000 999.000 999.000

DX 1.069 0.407 2.624 0.009

DY 1.227 0.523 2.345 0.019

Variances

TIME_RM_C 1.000 0.000 999.000 999.000

TIME_6M_C 1.000 0.000 999.000 999.000

X1 1.000 0.000 999.000 999.000

Y1 1.000 0.000 999.000 999.000

Residual Variances

X3 0.000 999.000 999.000 999.000

Y3 0.000 999.000 999.000 999.000

DX 0.420 0.042 10.018 0.000

DY 0.578 0.047 12.312 0.000

STDY Standardization

Two-Tailed

Estimate S.E. Est./S.E. P-Value

DX BY

X3 1.428 0.074 19.177 0.000

DY BY

Y3 1.302 0.056 23.382 0.000

DY ON

X1 -0.015 0.058 -0.266 0.790

Y1 -0.628 0.038 -16.343 0.000

TIME_6M_C -0.086 0.051 -1.693 0.090

TIME_RM_C 0.032 0.052 0.622 0.534

DX ON

Y1 -0.032 0.048 -0.668 0.504

X1 -0.657 0.036 -18.460 0.000

TIME_6M_C -0.240 0.043 -5.551 0.000

TIME_RM_C 0.097 0.040 2.412 0.016

X3 ON

X1 1.121 0.053 21.008 0.000

Y3 ON

Y1 0.905 0.045 20.215 0.000

DX WITH

DY 0.141 0.075 1.865 0.062

X1 WITH

Y1 0.323 0.054 5.928 0.000

TIME_RM_C -0.085 0.053 -1.598 0.110

TIME_6M_C 0.173 0.083 2.093 0.036

Y1 WITH

TIME_RM_C -0.128 0.056 -2.300 0.021

TIME_6M_C 0.063 0.075 0.842 0.400

TIME_6M_ WITH

TIME_RM_C 0.011 0.065 0.172 0.864

Means

TIME_RM_C 7.790 0.507 15.361 0.000

TIME_6M_C 5.995 0.224 26.731 0.000

X1 1.056 0.062 16.988 0.000

Y1 1.796 0.069 26.029 0.000

Intercepts

X3 0.000 0.000 999.000 999.000

Y3 0.000 0.000 999.000 999.000

DX 1.069 0.407 2.624 0.009

DY 1.227 0.523 2.345 0.019

Variances

TIME_RM_C 1.000 0.000 999.000 999.000

TIME_6M_C 1.000 0.000 999.000 999.000

X1 1.000 0.000 999.000 999.000

Y1 1.000 0.000 999.000 999.000

Residual Variances

X3 999.000 999.000 999.000 999.000

Y3 999.000 999.000 999.000 999.000

DX 0.420 0.042 10.018 0.000

DY 0.578 0.047 12.312 0.000

STD Standardization

Two-Tailed

Estimate S.E. Est./S.E. P-Value

DX BY

X3 1.075 0.052 20.489 0.000

DY BY

Y3 1.191 0.053 22.305 0.000

DY ON

X1 -0.018 0.069 -0.266 0.790

Y1 -0.758 0.044 -17.164 0.000

TIME_6M_C -0.041 0.024 -1.703 0.088

TIME_RM_C 0.026 0.042 0.621 0.535

DX ON

Y1 -0.039 0.058 -0.669 0.503

X1 -0.778 0.042 -18.375 0.000

TIME_6M_C -0.113 0.020 -5.749 0.000

TIME_RM_C 0.078 0.032 2.423 0.015

X3 ON

X1 1.000 0.000 999.000 999.000

Y3 ON

Y1 1.000 0.000 999.000 999.000

DX WITH

DY 0.141 0.075 1.865 0.062

X1 WITH

Y1 0.225 0.042 5.408 0.000

TIME_RM_C -0.090 0.057 -1.572 0.116

TIME_6M_C 0.311 0.152 2.041 0.041

Y1 WITH

TIME_RM_C -0.132 0.059 -2.232 0.026

TIME_6M_C 0.111 0.133 0.833 0.405

TIME_6M_ WITH

TIME_RM_C 0.030 0.172 0.172 0.864

Means

TIME_RM_C 9.697 0.069 140.856 0.000

TIME_6M_C 12.726 0.148 86.251 0.000

X1 0.892 0.048 18.554 0.000

Y1 1.487 0.046 32.387 0.000

Intercepts

X3 0.000 0.000 999.000 999.000

Y3 0.000 0.000 999.000 999.000

DX 1.069 0.407 2.624 0.009

DY 1.227 0.523 2.345 0.019

Variances

TIME_RM_C 1.550 0.213 7.271 0.000

TIME_6M_C 4.506 0.369 12.213 0.000

X1 0.713 0.044 16.262 0.000

Y1 0.685 0.040 17.162 0.000

Residual Variances

X3 999.000 999.000 999.000 999.000

Y3 999.000 999.000 999.000 999.000

DX 0.420 0.042 10.018 0.000

DY 0.578 0.047 12.312 0.000

R-SQUARE

Observed Two-Tailed

Variable Estimate S.E. Est./S.E. P-Value

X3 1.000 999.000 999.000 999.000

Y3 1.000 999.000 999.000 999.000

Latent Two-Tailed

Variable Estimate S.E. Est./S.E. P-Value

DX 0.580 0.042 13.845 0.000

DY 0.422 0.047 8.983 0.000

CONFIDENCE INTERVALS OF MODEL RESULTS

Lower .5% Lower 2.5% Lower 5% Estimate Upper 5% Upper 2.5% Upper .5%

DX BY

X3 1.000 1.000 1.000 1.000 1.000 1.000 1.000

DY BY

Y3 1.000 1.000 1.000 1.000 1.000 1.000 1.000

DY ON

X1 -0.233 -0.183 -0.157 -0.022 0.113 0.139 0.190

Y1 -1.101 -1.054 -1.030 -0.904 -0.777 -0.753 -0.706

TIME_6M_C -0.122 -0.105 -0.096 -0.048 -0.001 0.008 0.025

TIME_RM_C -0.098 -0.067 -0.051 0.031 0.113 0.129 0.159

DX ON

Y1 -0.203 -0.165 -0.145 -0.042 0.061 0.081 0.120

X1 -1.005 -0.964 -0.944 -0.837 -0.730 -0.709 -0.669

TIME_6M_C -0.177 -0.164 -0.157 -0.122 -0.087 -0.080 -0.067

TIME_RM_C -0.005 0.016 0.027 0.084 0.140 0.151 0.172

X3 ON

X1 1.000 1.000 1.000 1.000 1.000 1.000 1.000

Y3 ON

Y1 1.000 1.000 1.000 1.000 1.000 1.000 1.000

DX WITH

DY -0.035 -0.005 0.010 0.089 0.168 0.183 0.212

X1 WITH

Y1 0.118 0.144 0.157 0.225 0.294 0.307 0.333

TIME_RM_C -0.236 -0.201 -0.183 -0.090 0.004 0.022 0.057

TIME_6M_C -0.081 0.012 0.060 0.311 0.561 0.609 0.703

Y1 WITH

TIME_RM_C -0.285 -0.248 -0.229 -0.132 -0.035 -0.016 0.020

TIME_6M_C -0.231 -0.150 -0.108 0.111 0.329 0.371 0.453

TIME_6M_ WITH

TIME_RM_C -0.415 -0.308 -0.254 0.030 0.313 0.368 0.474

Means

TIME_RM_ 9.520 9.562 9.584 9.697 9.810 9.832 9.875

TIME_6M_ 12.346 12.437 12.484 12.726 12.969 13.016 13.106

X1 0.768 0.798 0.813 0.892 0.971 0.986 1.016

Y1 1.369 1.397 1.411 1.487 1.562 1.577 1.605

Intercepts

X3 0.000 0.000 0.000 0.000 0.000 0.000 0.000

Y3 0.000 0.000 0.000 0.000 0.000 0.000 0.000

DX 0.001 0.276 0.416 1.149 1.882 2.023 2.297

DY -0.167 0.223 0.422 1.462 2.502 2.701 3.090

Variances

TIME_RM_ 1.001 1.132 1.199 1.550 1.900 1.968 2.099

TIME_6M_ 3.556 3.783 3.899 4.506 5.113 5.230 5.457

X1 0.600 0.627 0.641 0.713 0.785 0.799 0.826

Y1 0.583 0.607 0.620 0.685 0.751 0.764 0.788

Residual Variances

X3 0.000 0.000 0.000 0.000 0.000 0.000 0.000

Y3 0.000 0.000 0.000 0.000 0.000 0.000 0.000

DX 0.383 0.407 0.420 0.486 0.551 0.564 0.588

DY 0.653 0.693 0.713 0.821 0.928 0.948 0.988

CONFIDENCE INTERVALS OF STANDARDIZED MODEL RESULTS

STDYX Standardization

Lower .5% Lower 2.5% Lower 5% Estimate Upper 5% Upper 2.5% Upper .5%

DX BY

X3 1.236 1.282 1.306 1.428 1.551 1.574 1.620

DY BY

Y3 1.159 1.193 1.210 1.302 1.394 1.411 1.445

DY ON

X1 -0.165 -0.129 -0.111 -0.015 0.080 0.099 0.134

Y1 -0.727 -0.703 -0.691 -0.628 -0.565 -0.553 -0.529

TIME_6M_C -0.218 -0.186 -0.170 -0.086 -0.002 0.014 0.045

TIME_RM_C -0.102 -0.070 -0.053 0.032 0.118 0.134 0.166

DX ON

Y1 -0.157 -0.127 -0.112 -0.032 0.047 0.062 0.092

X1 -0.749 -0.727 -0.715 -0.657 -0.598 -0.587 -0.565

TIME_6M_C -0.352 -0.325 -0.311 -0.240 -0.169 -0.155 -0.129

TIME_RM_C -0.007 0.018 0.031 0.097 0.163 0.176 0.201

X3 ON

X1 0.984 1.017 1.033 1.121 1.209 1.226 1.259

Y3 ON

Y1 0.790 0.817 0.831 0.905 0.978 0.993 1.020

DX WITH

DY -0.054 -0.007 0.017 0.141 0.265 0.288 0.335

X1 WITH

Y1 0.182 0.216 0.233 0.323 0.412 0.429 0.463

TIME_RM_C -0.223 -0.190 -0.173 -0.085 0.003 0.019 0.052

TIME_6M_C -0.040 0.011 0.037 0.173 0.310 0.336 0.387

Y1 WITH

TIME_RM_C -0.272 -0.237 -0.220 -0.128 -0.037 -0.019 0.015

TIME_6M_C -0.130 -0.084 -0.060 0.063 0.186 0.209 0.255

TIME_6M_ WITH

TIME_RM_C -0.157 -0.117 -0.096 0.011 0.119 0.139 0.179

Means

TIME_RM_ 6.483 6.796 6.955 7.790 8.624 8.784 9.096

TIME_6M_ 5.417 5.555 5.626 5.995 6.364 6.435 6.573

X1 0.896 0.934 0.954 1.056 1.159 1.178 1.217

Y1 1.618 1.661 1.683 1.796 1.910 1.931 1.974

Intercepts

X3 0.000 0.000 0.000 0.000 0.000 0.000 0.000

Y3 0.000 0.000 0.000 0.000 0.000 0.000 0.000

DX 0.020 0.271 0.399 1.069 1.738 1.867 2.117

DY -0.121 0.201 0.366 1.227 2.088 2.253 2.575

Variances

TIME_RM_ 1.000 1.000 1.000 1.000 1.000 1.000 1.000

TIME_6M_ 1.000 1.000 1.000 1.000 1.000 1.000 1.000

X1 1.000 1.000 1.000 1.000 1.000 1.000 1.000

Y1 1.000 1.000 1.000 1.000 1.000 1.000 1.000

Residual Variances

X3 999.000 999.000 999.000 0.000 999.000 999.000 999.000

Y3 999.000 999.000 999.000 0.000 999.000 999.000 999.000

DX 0.312 0.338 0.351 0.420 0.489 0.502 0.528

DY 0.457 0.486 0.501 0.578 0.655 0.670 0.699

STDY Standardization

Lower .5% Lower 2.5% Lower 5% Estimate Upper 5% Upper 2.5% Upper .5%

DX BY

X3 1.236 1.282 1.306 1.428 1.551 1.574 1.620

DY BY

Y3 1.159 1.193 1.210 1.302 1.394 1.411 1.445

DY ON

X1 -0.165 -0.129 -0.111 -0.015 0.080 0.099 0.134

Y1 -0.727 -0.703 -0.691 -0.628 -0.565 -0.553 -0.529

TIME_6M_C -0.218 -0.186 -0.170 -0.086 -0.002 0.014 0.045

TIME_RM_C -0.102 -0.070 -0.053 0.032 0.118 0.134 0.166

DX ON

Y1 -0.157 -0.127 -0.112 -0.032 0.047 0.062 0.092

X1 -0.749 -0.727 -0.715 -0.657 -0.598 -0.587 -0.565

TIME_6M_C -0.352 -0.325 -0.311 -0.240 -0.169 -0.155 -0.129

TIME_RM_C -0.007 0.018 0.031 0.097 0.163 0.176 0.201

X3 ON

X1 0.984 1.017 1.033 1.121 1.209 1.226 1.259

Y3 ON

Y1 0.790 0.817 0.831 0.905 0.978 0.993 1.020

DX WITH

DY -0.054 -0.007 0.017 0.141 0.265 0.288 0.335

X1 WITH

Y1 0.182 0.216 0.233 0.323 0.412 0.429 0.463

TIME_RM_C -0.223 -0.190 -0.173 -0.085 0.003 0.019 0.052

TIME_6M_C -0.040 0.011 0.037 0.173 0.310 0.336 0.387

Y1 WITH

TIME_RM_C -0.272 -0.237 -0.220 -0.128 -0.037 -0.019 0.015

TIME_6M_C -0.130 -0.084 -0.060 0.063 0.186 0.209 0.255

TIME_6M_ WITH

TIME_RM_C -0.157 -0.117 -0.096 0.011 0.119 0.139 0.179

Means

TIME_RM_ 6.483 6.796 6.955 7.790 8.624 8.784 9.096

TIME_6M_ 5.417 5.555 5.626 5.995 6.364 6.435 6.573

X1 0.896 0.934 0.954 1.056 1.159 1.178 1.217

Y1 1.618 1.661 1.683 1.796 1.910 1.931 1.974

Intercepts

X3 0.000 0.000 0.000 0.000 0.000 0.000 0.000

Y3 0.000 0.000 0.000 0.000 0.000 0.000 0.000

DX 0.020 0.271 0.399 1.069 1.738 1.867 2.117

DY -0.121 0.201 0.366 1.227 2.088 2.253 2.575

Variances

TIME_RM_ 1.000 1.000 1.000 1.000 1.000 1.000 1.000

TIME_6M_ 1.000 1.000 1.000 1.000 1.000 1.000 1.000

X1 1.000 1.000 1.000 1.000 1.000 1.000 1.000

Y1 1.000 1.000 1.000 1.000 1.000 1.000 1.000

Residual Variances

X3 999.000 999.000 999.000 999.000 999.000 999.000 999.000

Y3 999.000 999.000 999.000 999.000 999.000 999.000 999.000

DX 0.312 0.338 0.351 0.420 0.489 0.502 0.528

DY 0.457 0.486 0.501 0.578 0.655 0.670 0.699

STD Standardization

Lower .5% Lower 2.5% Lower 5% Estimate Upper 5% Upper 2.5% Upper .5%

DX BY

X3 0.940 0.973 0.989 1.075 1.162 1.178 1.211

DY BY

Y3 1.054 1.087 1.103 1.191 1.279 1.296 1.329

DY ON

X1 -0.196 -0.153 -0.132 -0.018 0.095 0.117 0.159

Y1 -0.872 -0.845 -0.831 -0.758 -0.686 -0.672 -0.645

TIME_6M_C -0.102 -0.087 -0.080 -0.041 -0.001 0.006 0.021

TIME_RM_C -0.082 -0.056 -0.043 0.026 0.095 0.108 0.134

DX ON

Y1 -0.189 -0.153 -0.135 -0.039 0.057 0.075 0.111

X1 -0.887 -0.861 -0.848 -0.778 -0.708 -0.695 -0.669

TIME_6M_C -0.164 -0.152 -0.146 -0.113 -0.081 -0.075 -0.062

TIME_RM_C -0.005 0.015 0.025 0.078 0.131 0.141 0.161

X3 ON

X1 1.000 1.000 1.000 1.000 1.000 1.000 1.000

Y3 ON

Y1 1.000 1.000 1.000 1.000 1.000 1.000 1.000

DX WITH

DY -0.054 -0.007 0.017 0.141 0.265 0.288 0.335

X1 WITH

Y1 0.118 0.144 0.157 0.225 0.294 0.307 0.333

TIME_RM_C -0.236 -0.201 -0.183 -0.090 0.004 0.022 0.057

TIME_6M_C -0.081 0.012 0.060 0.311 0.561 0.609 0.703

Y1 WITH

TIME_RM_C -0.285 -0.248 -0.229 -0.132 -0.035 -0.016 0.020

TIME_6M_C -0.231 -0.150 -0.108 0.111 0.329 0.371 0.453

TIME_6M_ WITH

TIME_RM_C -0.415 -0.308 -0.254 0.030 0.313 0.368 0.474

Means

TIME_RM_ 9.520 9.562 9.584 9.697 9.810 9.832 9.875

TIME_6M_ 12.346 12.437 12.484 12.726 12.969 13.016 13.106

X1 0.768 0.798 0.813 0.892 0.971 0.986 1.016

Y1 1.369 1.397 1.411 1.487 1.562 1.577 1.605

Intercepts

X3 0.000 0.000 0.000 0.000 0.000 0.000 0.000

Y3 0.000 0.000 0.000 0.000 0.000 0.000 0.000

DX 0.020 0.271 0.399 1.069 1.738 1.867 2.117

DY -0.121 0.201 0.366 1.227 2.088 2.253 2.575

Variances

TIME_RM_ 1.001 1.132 1.199 1.550 1.900 1.968 2.099

TIME_6M_ 3.556 3.783 3.899 4.506 5.113 5.230 5.457

X1 0.600 0.627 0.641 0.713 0.785 0.799 0.826

Y1 0.583 0.607 0.620 0.685 0.751 0.764 0.788

Residual Variances

X3 999.000 999.000 999.000 999.000 999.000 999.000 999.000

Y3 999.000 999.000 999.000 999.000 999.000 999.000 999.000

DX 0.312 0.338 0.351 0.420 0.489 0.502 0.528

DY 0.457 0.486 0.501 0.578 0.655 0.670 0.699

SAMPLE STATISTICS FOR ESTIMATED FACTOR SCORES

SAMPLE STATISTICS

Means

DX DX_SE DY DY_SE

________ ________ ________ ________

-0.396 0.393 -0.218 0.427

Covariances

DX DX_SE DY DY_SE

________ ________ ________ ________

DX 0.855

DX_SE 0.015 0.147

DY 0.306 0.029 1.035

DY_SE 0.014 0.157 0.025 0.202

Correlations

DX DX_SE DY DY_SE

________ ________ ________ ________

DX 1.000

DX_SE 0.043 1.000

DY 0.325 0.074 1.000

DY_SE 0.034 0.912 0.054 1.000

PLOT INFORMATION

The following plots are available:

Histograms (sample values, estimated factor scores, estimated values, residuals)

Scatterplots (sample values, estimated factor scores, estimated values, residuals)

Latent variable distribution plots

DIAGRAM INFORMATION

Use View Diagram under the Diagram menu in the Mplus Editor to view the diagram.

If running Mplus from the Mplus Diagrammer, the diagram opens automatically.

Diagram output

c:\users\hugoc\dropbox\trabalhos\aline -posdoc\cortisol\time\final.dgm

Beginning Time: 09:49:20

Ending Time: 09:49:20

Elapsed Time: 00:00:00

MUTHEN & MUTHEN

3463 Stoner Ave.

Los Angeles, CA 90066

Tel: (310) 391-9971

Fax: (310) 391-8971

Web: www.StatModel.com

Support: Support@StatModel.com

Copyright (c) 1998-2021 Muthen & Muthen
